# Chemical interactions in composites of gellan gum and bioactive glass: self-crosslinking and *in vitro* dissolution

**DOI:** 10.3389/fchem.2023.1133374

**Published:** 2023-05-12

**Authors:** A. Astanina, J. T. Koivisto, M. Hannula, T. Salminen, M. Kellomäki, J. Massera

**Affiliations:** ^1^ Faculty of Medicine and Health Technology, Tampere University, Tampere, Finland; ^2^ Division of Pathology, Department of Laboratory Medicine, Karolinska Institutet, Stockholm, Sweden; ^3^ Tampere Microscopy Center, Faculty of Engineering and Natural Sciences, Tampere University, Tampere, Finland

**Keywords:** bioactive glass, gellan gum, composite, physical crosslinking, organic–inorganic interactions, biodegradation, lysozyme, mechanical properties

## Abstract

We investigated the interactions between the organic–inorganic phases in composites and the impact on *in vitro* dissolution. The composite consists of a hydrogel-forming polysaccharide gellan gum (GG, organic phase) and a borosilicate bioactive glass (BAG, inorganic phase). The BAG loading in the gellan gum matrix varied from 10 to 50 wt%. While mixing GG and BAG, the ions released from BAG microparticles crosslinked with the carboxylate anions of GG. The nature of the crosslinking was assessed, and its impact on mechanical properties, swelling ratio, and enzymatic degradation profile upon immersion for up to 2 weeks was studied. Loading up to 30 wt% of BAG in GG caused an increase in mechanical properties associated with an increasing crosslinking density. At higher BAG loading, excess divalent ions and percolation of particles led to a decrease in the fracture strength and compressive modulus. Upon immersion, a decrease in the composite mechanical properties was attributed to the dissolution of the BAG and the loosening of the glass/matrix interface. The enzymatic degradation of the composites was inhibited at higher BAG loadings (40 and 50 wt%) even when the specimen was immersed for 48 h in PBS buffer with lysozyme. During *in vitro* dissolution in both SBF and PBS, the ions released from the glass led to the precipitation of hydroxyapatite already at day 7. In conclusion, we thoroughly discussed the *in vitro* stability of the GG/BAG composite and established the maximum BAG loading to enhance the GG crosslinking and mechanical properties. Based on this study, 30, 40, and 50 wt% of BAG in GG will be further investigated in an *in vitro* cell culture study.

## Graphical Abstract

**Figure:**
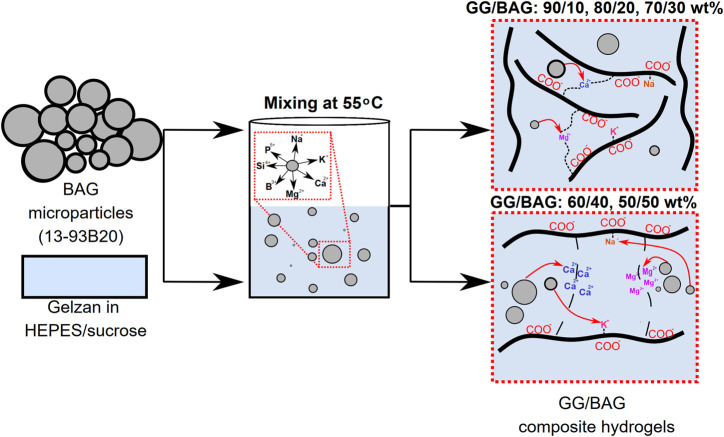
Schematic of the crosslinking process of gellan gum by Ca^2+^ and Mg^2+^ released from bioactive glass.

## 1 Introduction

At present, there is a growing interest in natural polysaccharide-based composite hydrogels in the field of biomaterials (MuhamadLazim et al., 2019). Plant-, animal-, and microorganism-derived polysaccharides, such as alginate, cellulose, chitosan, hyaluronic acid, and gellan gum, are investigated in various scientific publications (Jayakumar et al., 2010; Moon et al., 2011; Koivisto et al., 2017; Bukhari et al., 2018; Neves et al., 2020). Composites based on polysaccharides [e.g., hyaluronan, chitosan, and alginate crosslinked with a bioactive glass (BAG)] were investigated as potential bone grafts (Zhao et al., 2016; Martins et al., 2017; Sikkema et al., 2021). Polysaccharides are physically crosslinked with cations leading to the formation of hydrogels (Alves et al., 2021). Polysaccharide-based composites are physically crosslinked with ions released from BAG during processing. In all those composites, the presence of BAG impacted the mechanical properties and the swelling response of the material. In this study, we focused our effort on understanding the chemical interactions between gellan gum (GG) and BAG.

GG is an anionic polysaccharide derived from *Sphingomonas* (formerly *Pseudomonas*) *elodea*, and it consists of repeating units of polysaccharides: 2x β-D-glucose, *ß*-D-glucuronic acid, and α-L-rhamnose (Morris et al., 2012). The ability of GG to crosslink with divalent ions was first introduced in the early 1980s (Kang et al., 1982; Grasdalen and Smidsrød, 1987; Morris et al., 2012). Tang et al. proved that GGs crosslinked with Ca^2+^ are stronger than those crosslinked with Mg^2+^ (Tang et al., 1995). These results are of particular interest for the development of composites. Indeed, Douglas et al. stated that GG hydrogels self-crosslinked in the presence of divalent cations dissolved from BAG during processing (Douglas et al., 2014). Divalent cations released from BAG bind to carboxylate anions (crosslinking sites) from GG, leading to the formation of crosslinks. The ratio between ions released and crosslinking sites impacts the strength of the composite and enzymatic attack on glycosidic bonds in GG/BAG (Xu et al., 2018). The number of free crosslinking sites in GG/BAG impacts the interaction of the composite with water molecules and subsequently the hydrolytic degradation of the composite.

GG is well-known to be biocompatible, biodegradable, and easy to functionalize, and was suggested as a material for scaffold development in tissue engineering. GG has been studied for bone, cartilage, and spinal cord tissue engineering applications (Smith et al., 2007; Stevens et al., 2016). Our group has previously shown the suitability of GG for neurons differentiated from human embryonic stem cells and human-induced pluripotent stem cells (Koivisto et al., 2017), as well as for bone differentiation of human adipose stem cells (Vuornos et al., 2019). Good biocompatibility was demonstrated with both cell types.

In addition, we have previously studied 13-93B20 in combination with gelatin and with pre-osteoblastic MC3T3-E1 cells. It was demonstrated that cells spread, attach, and proliferate on hybrid biomaterials containing 13-93B20 (Houaoui et al., 2021).

In our work, we combined a polymeric organic phase of GG (matrix) and an inorganic phase of borosilicate BAG 13-93B20 (filler) in five different weight ratios to produce bioactive hydrogel composites. We hypothesize that the addition of BAG microparticles to GG is influencing the mechanical properties of GG/BAG composites, and that we would observe a local maximum within the loading ratio range studied. We also expected to see the effect of released BAG ions on the crosslinking of GG. From past experiments, one can also hypothesize that the inclusion of bioactive glass particles will decrease the enzymatic degradation rate. While cell culture is not the scope of this manuscript, it is expected that the addition of 13-93B20 promotes the bioactivity of the composite due to the fast formation of a calcium–phosphate-rich layer on its surface during incubation in aqueous media. This should promote osteoblast cell adhesion, proliferation, and osteogenic differentiation (Vuornos et al., 2019; Houaoui et al., 2021). Here, the aim is to better understand the interactions between the organic and inorganic phases to tailor the mechanical properties and *in vitro* dissolution (in PBS, lysozyme/PBS, and SBF), while guaranteeing the precipitation of a calcium–phosphate reactive layer, which is perceived as the first sign of bioactivity.

## 2 Materials and methods

### 2.1 Preparation of samples and evaluation of interactions between organic and inorganic phases

#### 2.1.1 Preparation of GG samples with BAG

GG was prepared in a HEPES/Sucrose buffer (25 mM, pH = 6.45–6.50/sucrose 10% w/w) (Beynon, 2020). First, HEPES (Sigma-Aldrich, Co., St. Louis, USA) was dissolved in 100 ml of ultrapure water to create the HEPES/sucrose buffer, which will, hypothetically, be used with cells as well. 25 mM HEPES/sucrose buffer is used to maintain the ion concentration at biological pH (Naddaf Dezfuli et al., 2014). After adjusting the pH, sucrose (Sigma-Aldrich, Co., St. Louis, USA) was added to the solution in deionized water (Koivisto et al., 2017). The buffer solution was sterilized using a PALL Acrodisc PF 32-mm syringe filter with 0.8/0.2 µm Supor membrane (Agilent Technologies). Finally, GG (Gelzan^®^, Sigma-Aldrich, Co., St. Louis, USA) was dissolved in HEPES/sucrose buffer at 70°C at a GG concentration of 5 mg/ml (Koivisto et al., 2017).

The inorganic phase (borosilicate BAG) used in this study was 13-93B20 (composition in mol%: 43.7SiO_2_-10.9B_2_O_3_-22.1CaO-7.9K_2_O-7.7MgO-6.0Na_2_O-1.7P_2_O_5_), and its preparation protocol has been reported in detail in the work of Houaoui et al. (2021). BAG was milled and sieved to particle sizes <38 µm.

The BAG 13-93B20 particles were mixed with GG containing HEPES/sucrose buffer at 55°C. We prepared composite GG/BAG hydrogels with weight ratios of 90/10, 80/20, 70/30, 60/40, and 50/50 wt%. A 4.5 ml GG solution was initially mixed in a beaker for 10 min, after which we added the BAG particles and continued mixing the GG solution with the particles for another 10 min at a temperature of 55°C and at 250 rpm. Then, the mixture was cast in 0.65-ml molds. Hydrogels were stored in the humidity chamber (humidity level of 70%) at room temperature overnight to guarantee complete hydrogel gelation and then used for further testing. Additionally, control samples with the same amount of BAG but without GG were prepared in HEPES/sucrose buffer, and ion release was assessed.

#### 2.1.2 Calculation of possible crosslinking sites in GG for interactions with ions released from borosilicate BAG

To evaluate how various BAG loading affects the crosslinking of the GG, we calculated the amount of crosslinking sites per GG in the stated weight ratios between GG and BAG: 90/10, 80/20, 70/30, 60/40, and 50/50 wt%. Gelzan® is a deacylated form of GG, which is composed of repeating units (monomers) of 2×β-D-glucose, *β*-D-glucuronic acid, and α-L-rhamnose. Each monomer has a crosslinking site with a carboxylate anion of *β*-D-glucuronic acid, which forms an ionic bonding with monovalent and divalent metals. Specifically, the bonding occurs with alkaline and alkaline earth metal cations such as Na^+^, K^+^, Ca^2+^, and Mg^2+^ (Morris et al., 2012). To estimate the amount of crosslinking sites, we calculated the amount of repeating units (n) in 100 wt% of GG (Eq. 1).

$$n=\frac{M_{gg}}{M\;one\;repeating\;unit},$$ (1)

n —the amount of repeating units; $M_{gg}$ —molar mass of GG; M one repeating unit—a molar mass of 2xβ-D-glucose, *ß*-D-glucuronic acid, and α-L-rhamnose

$$n=\frac{500\;000\;g/mol}{715\;g/mol}=699.3\;∼699.$$

As can be seen from Eq. 1, in a pure (100 wt%) GG, there are 699 repeating units, which implies that there are 699 crosslinking sites.

We hypothesized that crosslinks are formed between carboxylate anions of GG and divalent cations from BAG (Ca^2+^ and Mg^2+^), one divalent ion per two crosslinking sites (or one crosslink). We did not calculate monovalent Na^+^ and K^+^ ions for the formation of possible crosslinks with GG due to the reaction of these ions with HEPES, glucose, and fructose at acidic pH = 6.45–6.50 of HEPES/sucrose buffer (as products of hydrolyzed sucrose) with further formation of salts (Buttersack et al., 2021).

The GG/BAG compositions used in this work are presented in Table 4, along with their calculated crosslinking sites and measured gelation time (Table 1).

**TABLE 1:** Gelation time of GG/BAG samples (tube tilt test).

GG/BAG, wt%	90/10 (min)	80/20	70/30	60/40	50/50 (min)
**Gelation time**	5	3 min 30 s	2 min 30 s	2 min 30 s	1

#### 2.1.3 Estimation of gelation time

To assess the gelation time of the GG/BAG samples with various wt% BAG (as mentioned in Table 1), we used a 5-ml cut syringe as a mold. Furthermore, the gelation time was estimated with the tube tilt test (Tanodekaew et al., 2003). After mixing, a 0.65-ml heated (up to 55 °C) mixture of GG dissolved in HEPES/sucrose buffer with different concentrations of BAG was poured into the syringe mold. Then, the mold was tilted slowly every 30 s to assess the flow of the gel. If the mixture was still in a liquid state once the mold was titled, the tilting test was repeated after 30 s. Once the solution gelated and did not flow while tilting, the gelation time was recorded. The gelation time of each wt% of BAG in GG is presented in Table 1.

### 2.2 Chemical and physical properties of the composites

#### 2.2.1 Evaluation of inorganic content

The evaluation of inorganic phase loading was performed by the calcination test (ISO 1172). Samples were placed into dried porcelain crucibles. The crucibles were heated up to 625°C for 10 min and cooled to ambient temperature in a glass desiccator to dry the crucibles. The process was repeated until the difference in the crucible’s mass before and after the drying step was smaller than 0.3–0.4 mg. The mass of the dried crucible was recorded as m_1_. The hydrogels were placed into the dry crucibles and covered with punched aluminum foil and parafilm. The crucibles containing the samples were frozen overnight at −20°C and then freeze-dried for 20 h (Christ Epsilon 2–4). The freeze-dried samples were then heated at 105°C for 60 min in the ventilation oven (VWR, Venti-Line). The mass of the crucible with the sample after freezing, freeze-drying, and heating at the ventilation oven was recorded as m_2_. Then, the crucible with the dried sample was placed into the furnace (Nabertherm, P 330), and the organic part was burned off at 625°C for another 60 min. Calculations of the inorganic phase, in the composites, after calcination, are presented in Eq. 2:

$$\begin{array}{c} M_{residue}=\frac{m_{3}-m_{1}}{m_{2}-m_{1}}\cdot 100 \end{array},$$ (2)

where $m_{1}$ is the initial mass of the dry crucible, $m_{2}$ is the mass of the freeze-dried sample after heating up to 105°C in the ventilation oven, and $m_{3}$ is the final mass of the crucible plus residue after the calcination of the organic part. The measurements were reproduced on five samples per condition. The residual mass is reported as an average ± standard deviation (Eq. 3).

$$m_{BAG\;sample}=\frac{C_{GG}\times V_{vial}\times wt{\%}_{BAG}}{wt{\%}_{GG}}\times \frac{V_{sample}}{V_{vial}},$$ (3)

where $m_{BAG\;sample}$ is BAG loading per sample, $C_{GG}$ is the concentration of GG , $V_{vial}$ is the volume of the vial, $V_{sample}$ is the volume of one sample, and $wt{\%}_{BAG}$ , and $wt{\%}_{GG}$ are the ratios in weight percent of BAG and GG, respectively.

The mass of the GG in the sample was calculated using Eq. 4:

$$m_{GGsample}=C_{GG}\times \frac{V_{sample}}{V_{vial}}.$$ (4)

The mass of the sample was calculated using Eq. 5:

$$m_{sample}=m_{GG\;sample}+m_{BAGsample}.$$ (5)

#### 2.2.2 Electron microscopy

Cylindrical GG/BAG samples (day 0) were first freeze-dried and then embedded in epoxy resin. The cross-sections of the cylinders were polished before applying a 4-nm thick carbon layer before imaging. A Zeiss Crossbeam 540 scanning electron microscope equipped with an Oxford X-Max*N* 80 EDS detector was used to assess the presence of the particles and their composition.

#### 2.2.3 Thermal characterization

The thermal characterization of GG/BAG was carried out by thermogravimetric analysis (TGA) (TGA Q500 V6.7 Build 203) at a constant heating rate of 10°C/min, up to 1,000°C in a nitrogen atmosphere using an Al_2_O_3_ pan. The average mass of freeze-dried samples was 7.76 ± 0.83.

#### 2.2.4 FT-IR ATR

The samples were analyzed at room temperature using a Perkin Elmer FT-IR spectrometer (Spectrum Two FT-IR/Sp10 Software, Version 10.4.4., Perkin Elmer) in the spectral range from 600 to 4,000 cm^-1^. Analysis was conducted in the attenuated total reflectance mode. The resolution was 2 cm^-1^, and the results presented are the average of four scans.

#### 2.2.5 X-ray diffraction

The analysis was conducted using a PANalytical EMPYREAN Cu LFF HR X-Ray diffractometer. XRD measurements were performed between 2θ = 5° and 80°, at 40 mA and 45 kV, with step size 0.0263° and scan time 4.0 s per step.

#### 2.2.6 Compression test

Mechanical properties of the cylindrical GG/BAG samples were investigated in compression using BOSE Biodynamic test instrument (WinTest^®^ version 8.2, Waters Corporation, USA). The average height of the samples was 6.65 ± 0.3 mm, and the average diameter was 11.49 ± 0.22 mm. The mechanical properties were reproduced on five parallel samples. The speed of compression was set at 10 mm/min. The samples were compressed to 65% of the original height. The compressive modulus was calculated from the stress‐strain curve as the slope of the apparent region of linear elasticity. Fracture strength and fracture strain were analyzed as projections on the x- and y-axes from the peak of the curve (Figure 3C, Supplementary Material S1), respectively. Statistical significance between the mean ranks for equal distributions was assessed by a *t*-test. Statistical significance is taken for values of *p* < 0.05. The experimental results are illustrated as means ± standard deviation.

### 2.3 *In vitro* dissolution studies

*In vitro* dissolution studies were conducted in three media: 1) phosphate-buffered saline (PBS) to assess the hydrolytic degradation profile, 2) lysozyme in PBS to assess the enzymatic degradation profile, and 3) simulated body fluid (SBF) (Kokubo, 1991) to assess the bioactivity (i.e., precipitation of hydroxyapatite upon immersion) of the composites. All samples were gelated overnight in a humidity chamber at room temperature before adding the liquid for *in vitro* dissolution tests.

#### 2.3.1 *In vitro* dissolution in PBS

PBS was prepared using NaCl, NaH_2_PO_4_x2H_2_O (VWR CHEMICALS, Leuven, Belgium), and Na_2_HPO_4_xH_2_O (J.T. Baker, Deventer, Holland). The pH measured on the following day was in the range of 7.35–7.45. The initial volume ratio between the sample and PBS was constant during the dissolution series. 90/10, 80/20, 70/30, 60/40, and 50/50 wt% GG/BAG samples were investigated *in vitro* PBS. The samples were studied in five replicates per condition and kept in an orbital incubator (INFORS Multitron II) at 37°C and 100 rpm. At 1, 2, 3, 7, and 14 days, the pH was measured using an S47-K Seven Multi™ pH-meter (Mettler-Toledo LLC, Ohio, USA). 1 ml of the solution was taken and replaced from stock PBS and diluted in 9 ml of (1M) nitric acid (CAS7697-37-2 Romil Ltd.). Elemental concentrations of ions in the solution were measured using Agilent technologies 55110 Inductively Coupled Plasma Optical Emission Spectrometer (ICP-OES) equipped with an autosampler. ICP standards of the applicable elements were all purchased from Romil Ltd. Phosphorus (Phosphorus 100 ppm PrimAg, solute: NH_4_H_2_PO_4_, matrix HNO_3_), calcium (Calcium 100 ppm PrimAg, solute: CaCO_3_, matrix: HNO_3_), and sodium (Sodium 100 ppm PrimAg, solute: Na_2_CO_3_, matrix: HNO_3_). Standards were diluted to 4, 10, 20, and 40 ppm to obtain a calibration curve. Wavelengths for analysis used were 288.158 ppm (Si), 249.772 ppm (B), 393.366 ppm (Ca), 766.491 ppm (K), and 280.270 ppm (Mg). At days 1, 2, 3, 7, and 14 of immersion in PBS, the mechanical properties were measured in compression as described previously.

##### 2.3.1.1 Swelling ratio

The swelling ratio was measured after immersing the samples in 2.5 ml of PBS (prepared as described in 2.3.1) for 1, 2, 3, 7, and 14 days. The hydrogels were weighted before immersion (w_0_) and post immersion (w_s_). The wet swelling ratio (S) was calculated using Eq. 6 (Douglas et al., 2014):

$$S=\frac{w_{s}-w_{o}}{w_{o}}\times 100.$$ (6)

#### 2.3.2 Enzymatic degradation

The stability of samples was assessed by immersing them in lysozyme/PBS solution (3 mg/ml) for 1, 2, 4, 6, 8, 24, and 48 h. Lysozyme is an antibacterial enzyme that breaks down the cell walls and breaks the ether bonds connecting the structural backbone of GG (Xu et al., 2018). The samples were also incubated in PBS as a control. Samples in lysozyme/PBS and PBS were placed in the shaking incubator at 37°C and 100 rpm. The post-immersion changes in mass, pH, and ion release were evaluated. Elemental concentrations of ions in the solution were measured by ICP-OES as in 2.3.1. Wavelengths for analysis used 288.158 ppm (Si), 249.678 ppm (B), 393.366 ppm (Ca), 769.897 ppm (K), and 280.270 ppm (Mg). The same amount of 13–93B20 BAG as in GG/BAG samples (Table 2) was immersed in PBS and lysozyme/PBS (3.0 mg/ml) for 24 and 48 h only, respectively. This process is necessary to compare the pH of the media and ion release from the BAG between BAG alone and GG/BAG samples in lysozyme/PBS and in PBS. Comparison between BAG and GG/BAG pH and ion release values were investigated to check what impact BAG has on GG degradation.

**TABLE 2:** BAG loading in the 4.5 ml GG batch and in the 0.65-ml GG sample.

GG/BAG wt%	BAG, mg per batch	GG, mg per sample	BAG mg per sample calculated	BAG mg per sample (calcination test)
90/10	2.5	3.25	0.36	0.81
80/20	5.63	3.25	0.81	1.22
70/30	9.43	3.25	1.39	1.36
60/40	15.0	3.25	2.17	1.81
50/50	22.5	3.25	3.25	2.95

#### 2.3.3 Bioactivity in SBF

SBF was prepared following the protocol described by Kokubo (1991). Each sample was immersed in the SBF solution at a fixed mass‐volume ratio of 1.5 mg BAG in sample/1 ml of SBF solution as suggested in Maçon et al. (2015). The samples were placed in a shaking incubator at 37°C and 100 rpm. During the experiment, the solution was not refreshed to evaluate the precipitation of the Ca–P layer. pH post-immersion and ion concentrations were measured. Wavelengths for analysis used were 250.690 ppm (Si), 249.678 ppm (B), 766.491 ppm (K), 279.800 ppm (Mg), 317.933 ppm (Ca), and 253.561 ppm (P).

##### 2.3.3.1 X-ray fluorescence

Micro X-ray fluorescence (micro-XRF) analysis was performed to look at the distribution of Ca and P on the sample. Micro-XRF was conducted on samples containing GG/BAG materials at day 0 and day 7 of immersion in SBF. The measurements were performed using Bruker M4 Tornado plus using a rhodium X-ray tube. The measurements were performed in a helium atmosphere to allow better detection of the relatively light elements, Ca and P, and to preserve the moisture of the samples. Sample drying was still observed during the measurements, so the measurement time was limited to approximately 1 h.

##### 2.3.3.2 X-ray microtomography (µCT)

The samples were imaged with Zeiss Xradia MicroXCT-400 (Zeiss, Pleasanton, CA, USA) micro-CT device. Imaging was performed with the source voltage of 80 kV and current of 125 µA. A total of 1,601 projections were taken with 360° rotation angle and 3 s exposure time. A ×4 objective was used with binning two, resulting in a pixel size of 5.64 µm. The reconstruction was performed with Zeiss XMReconstructor software. Image processing, analysis, and visualizations were performed with the Avizo 2020.2 software (Thermo Fisher Scientific, Waltham, MA, USA).

## 3 Results and discussion

### 3.1 Chemical and physical properties of the composites

#### 3.1.1 BAG loading and dispersion

The nominal wt% of BAG in the gel was set to 10, 20, 30, 40, and 50 wt%. The actual BAG loading was quantified by the calcination test and reported in Table 2. The BAG loading was found to be close to the nominal one for the samples with 10, 20, and 30 wt% of BAG particles. The BAG loading was lower than expected for samples with higher BAG content (40 and 50 wt% of BAG particles). This can be assigned to the large number of particles that can aggregate during the mixing and thus be more likely to sediment to the bottom of the vial.

#### 3.1.2 3D visualization, internal structure, and elemental analysis

**FIGURE 1:**
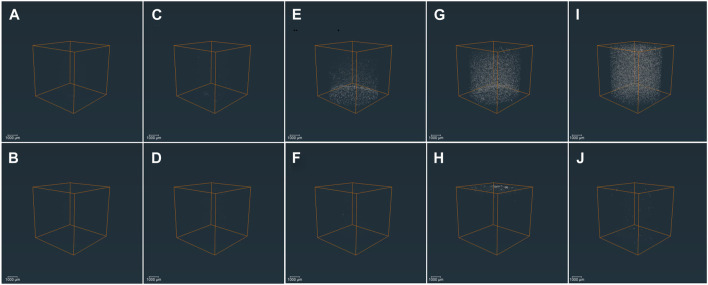
3D μCT images of the GG/BAG samples at day 0 (upper row) and day 7 (lower row) of immersion in SBF: **(A, B)** 90/10 wt%; **(C, D)** 80/20 wt%; **(E, F)** 70/30 wt%; **(G, H)** 60/40 wt%; and **(I, J)** 50/50 wt% GG/BAG.

The 3D μCT images of GG/BAG samples before and after 7 days of immersion in SBF are illustrated in Figure 1.

The particles’ distribution is visible in the GG/BAG 70/30, 60/40, and 50/50 wt% samples only before immersion (Figure 1). The particles are distributed uniformly in the GG/BAG 50/50 wt% samples but non-uniformly in the GG/BAG 60/40 wt% and GG/BAG 70/30 wt% samples. Dispersion of particles in GG/BAG 80/20 wt% and GG/BAG 90/10 wt% is not visible. The volume ratio of BAG in the sample was compared to the expected volume ratio of the BAG in the sample (Table 3) (Supplementary Figure S2).

**TABLE 3:** Amount and distribution BAG particles in GG/BAG samples (calculation from μCT images).

GG/BAG wt%	Mass (BAG/sample) (mg)		Volume (BAG/sample) (mm^3^)		Number of BAG particles per sample			Particles distribution in the sample	Diameter of particles minimum–maximum (µm)		Diameter of particles mean (µm)	
	Calculated	Measured	Calculated	Measured	Calculated	Measured day 0	Measured day 7 post immersion in SBF		Before immersion	Post immersion	Before immersion	Post immersion
90/10	0.36	0.62	0.035	0.00	153,111	954	1,663	Not visible	7–20	7–46	7.35	7.55
80/20	0.81	0.89	0.08	0.00	344,500	3,973	2,284	Not visible	7–55	7–41	10.39	7.39
70/30	1.39	1.36	0.13	0.07	599,179	24,635	749	Uniform in the half-bottom part, the presence of agglomerates	7–75	7–55	19.33	8.16
60/40	2.17	1.81	0.21	0.19	922,920	63,265	5,349	Uniform, except for the top, the presence of agglomerates	7–85	7–211	19.97	17.78
50/50	3.25	2.95	0.31	0.32	1,382,254	112,842	6,489	Uniform, presence of agglomerates	7–146	7–89	19.30	9.95

The SEM images (Supplementary Figures S1A, C, E, G, and Supplementary Material S2) show BAG particles of mostly 1 to 5 µm in diameter distributed throughout the GG. EDS maps (Supplementary Figures S1B, D, F, H) clearly show the Si-rich particles. However, as shown in Supplementary Figure S2, the distribution of particles is only local and does not provide a proper representation of particle distribution throughout the sample due to large inhomogeneities as shown in Figure 1.

#### 3.1.3 Thermogravimetric analysis

**FIGURE 2:**
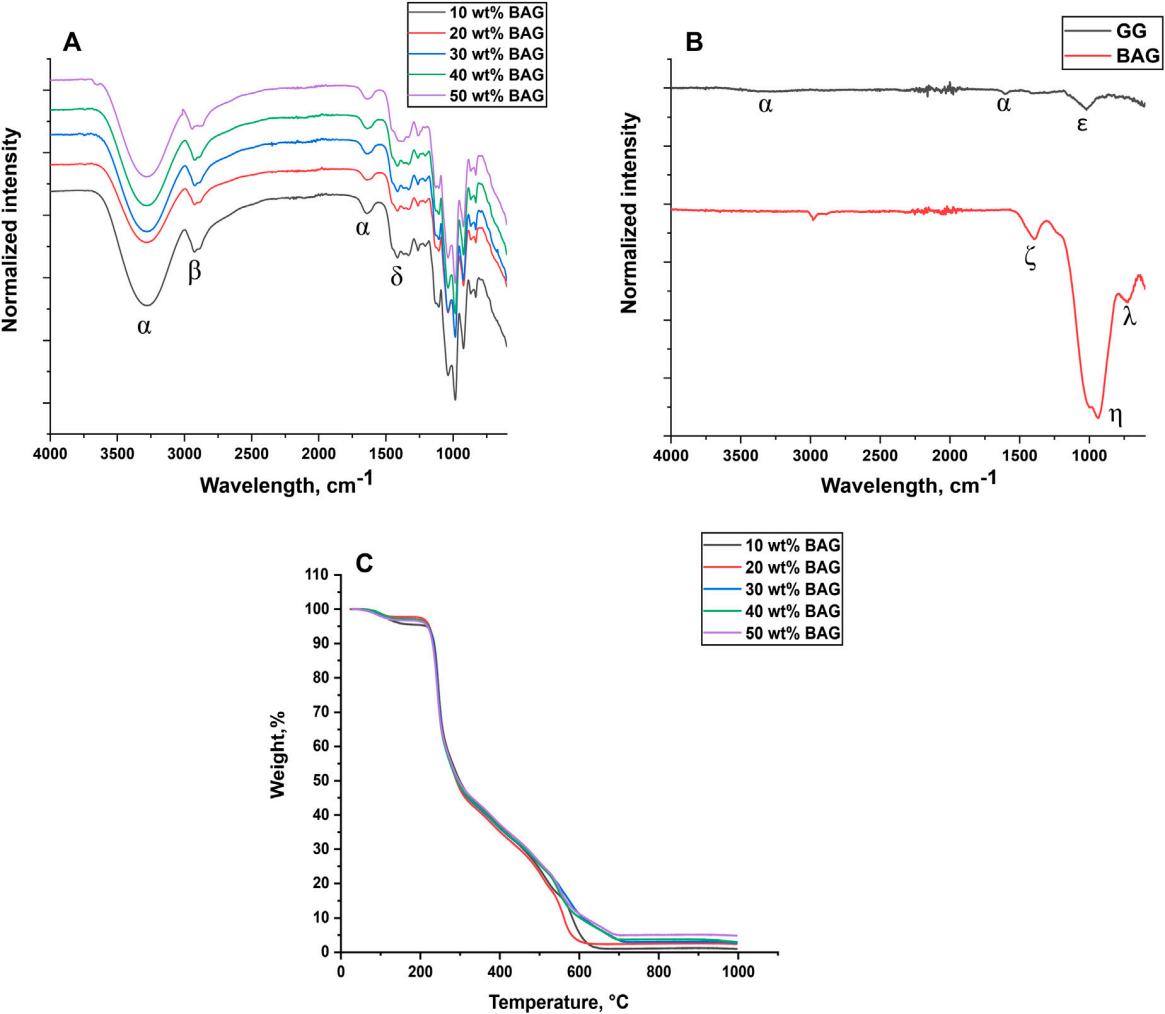
**(A, B)** FTIR-ATR Spectra of **(A)** GG/BAG; **(B)** raw GG and BAG and **(C)** thermogravimetric analysis of GG/BAG samples at day 0.

The thermal behavior of GG/BAG is illustrated in Figure 2C. The results indicate that the weight change of a sample occurs in three stages.

Initially, at 80–300°C, weight loss of GG/BAG is attributed to the evaporation of residual water and decomposition of GG. Residual water loss occurs between 80 and 220°C (Halim et al., 2012). Moreover, sucrose decomposes into caramel, furfural, and 5-hydroxymethylfurfural at a temperature above 160°C, and HEPES breaks down into piperazine and ethylene glycol at 238–242°C (Snyder and Sobocinski, 1975; Hurtta et al., 2004). During this stage, the weight significantly drops to 20–25% from the initial weight due to the decomposition of the polymer backbone and cleavage of glycosidic bonds into oligosaccharides and monosaccharides occurring at 200–250°C. Then, the oligosaccharides and monosaccharides decomposed into furans and acetic acid, respectively, at 250–300°C together with the loss of chemically bonded water from GG (Noor et al., 2012; Sukumar et al., 2021). This results in a weight loss of 50–53% from the initial weight.

In the second stage, between 300–700°C, the remaining fragments of GG are decomposed into smaller molecules (CO and CO_2_) showing a weight loss of 73–75% (Noor et al., 2012). 90/10 and 80/20 wt% GG/BAG had a rapid drop in mass at around 600°C, while GG/BAG 70/30, 60/40, and 50/50 wt% steadily decreased in mass until 700°C.

In the final stage, between 700–1,000°C, only a small amount of residue (2–5% of the initial weight) is left, which is mostly ash and BAG, indicating that no more compounds remain. The melting temperature of the BAG is expected to occur above 1,000°C (Houaoui et al., 2021) While TGA provides information on the thermal decomposition and calcination of the GG, the burning test should be preferred to quantify the amount of inorganic phase. Indeed, as shown in Figure 1, the glass distribution in the processed material is not homogeneous, and therefore, the light and small (7.76 ± 0.83 mg) samples used in TGA easily lead to significant errors when assessing the content of BAG microparticles. In the calcination tests, a larger sample (approximately 70 mg) is used, thus reducing the risk of selecting a sample volume with drastically different glass loading. Furthermore, when using TGA, the measured residual weight is overestimated due to the remnants of ash from the incomplete burning of the organic phase. Indeed, the residual weights at the last stage of TGA were measured, and the results are as follows: for 90/10 GG/BAG wt%: 0.14 mg, for 80/20 GG/BAG wt%: 0.25 mg, for 70/30 GG/BAG wt%: 0.33 mg, for GG/BAG 60/40 wt%: 0.37 mg, and for GG/BAG 50/50 wt%: 0.50 mg. However, the calculated weight of glass in the sample is lower than the residual weights measured: for 90/10 wt% GG/BAG: 0.04 mg, for 80/20 wt% GG/BAG: 0.08 mg, for 70/30 wt% GG/BAG: 0.15 mg, for 60/40 wt% GG/BAG: 0.23 mg, and for 50/50 wt% GG/BAG: 0.43 mg.

#### 3.1.4 FTIR-ATR and XRD characterization

The FTIR-ATR spectra and XRD patterns of raw GG powder, raw BAG, and lyophilized GG/BAG composites are illustrated in Figures 2A, B and Supplementary Figure S3.

The FTIR spectrum of raw GG exhibits -OH stretching peaks at ∼3,280 and ∼1,610 cm^-1^ (α) (Wenqiang et al., 2021), and –C-O vibration at ∼1,030 cm^-1^ (ε) (Leitermann et al., 2008), (Figure 2B). The XRD spectrum of GG presents one broad hallow at 2θ∼20° which refers to *ß*-D-glucose (Sponsler and Dore, 1931), and small sharp diffraction peaks at 2θ∼11° (α-L-rhamnose monohydrate) (McGeachin and Beevers, 1957), 2θ∼28°, and 2θ∼31° (β-D-glucose) (Sponsler and Dore, 1931). This implies that raw GG has some semi-crystalline structures of α-L-rhamnose and *ß*-D-glucose. Consequently, the –C-O vibration in the FTIR (Figure 2B) can be assigned to the L-rhamnose evidenced by XRD (Supplementary Figure S3).

The FTIR spectrum of BAG presents typical vibrations for borosilicate glass. The stretching of the B-O band in BO_3_ units is observed at ∼1,400 cm^-1^(ζ) (Figure 2B) (Kamistos and Chryssikos, 1991). The band observed at ∼930 cm^-1^ (η) can be attributed to Si-O^-^ (non-bridging oxygen) in the [SiO_4_] units (Lucovsky et al., 1983; Agathoupoulos et al., 2006), while the band found at ∼1,000 cm^-1^ (η) is related to Si-O-Si asymmetric stretching in the [SiO_4_] units (Domine and Piriou, 1983). The peak at ∼730 cm^-1^ (λ) is associated with the symmetric bending vibration of O-Si-O, (Agathoupoulos et al., 2006). As expected, the XRD spectrum of BAG only exhibits a broad band at 2θ∼30°, confirming the amorphousness of the glass.

The FTIR spectrum of GG/BAG is presented in Figure 2A. Broad -OH stretching peaks ∼3,280 cm^-1^ and ∼1,640 cm^-1^ (α) (Wenqiang et al., 2021); -CH and -CH_2_ stretching peaks ∼2,921–2,925 cm^-1^ (β) (Thombare et al., 2023); and C-H bending peak ∼1,410–1,414 cm^-1^ (δ) are visible in the spectra, as expected from GG hydrogels (Zhao and Wang, 2019; Rohallah and Mojde, 2021; Thombare et al., 2023). The X-ray diffraction patterns of the GG/BAG composites (Supplementary Figure S3) show two broad hallows between 2θ∼21° and 2θ∼39°, which suggests that all GG/BAG composites are amorphous. The dissolution of raw GG in HEPES/sucrose buffer and further polymerization of GG with ions released from BAG suppress the crystalline phases from the Gelzan raw material.

#### 3.1.5 Mechanical properties

**FIGURE 3:**
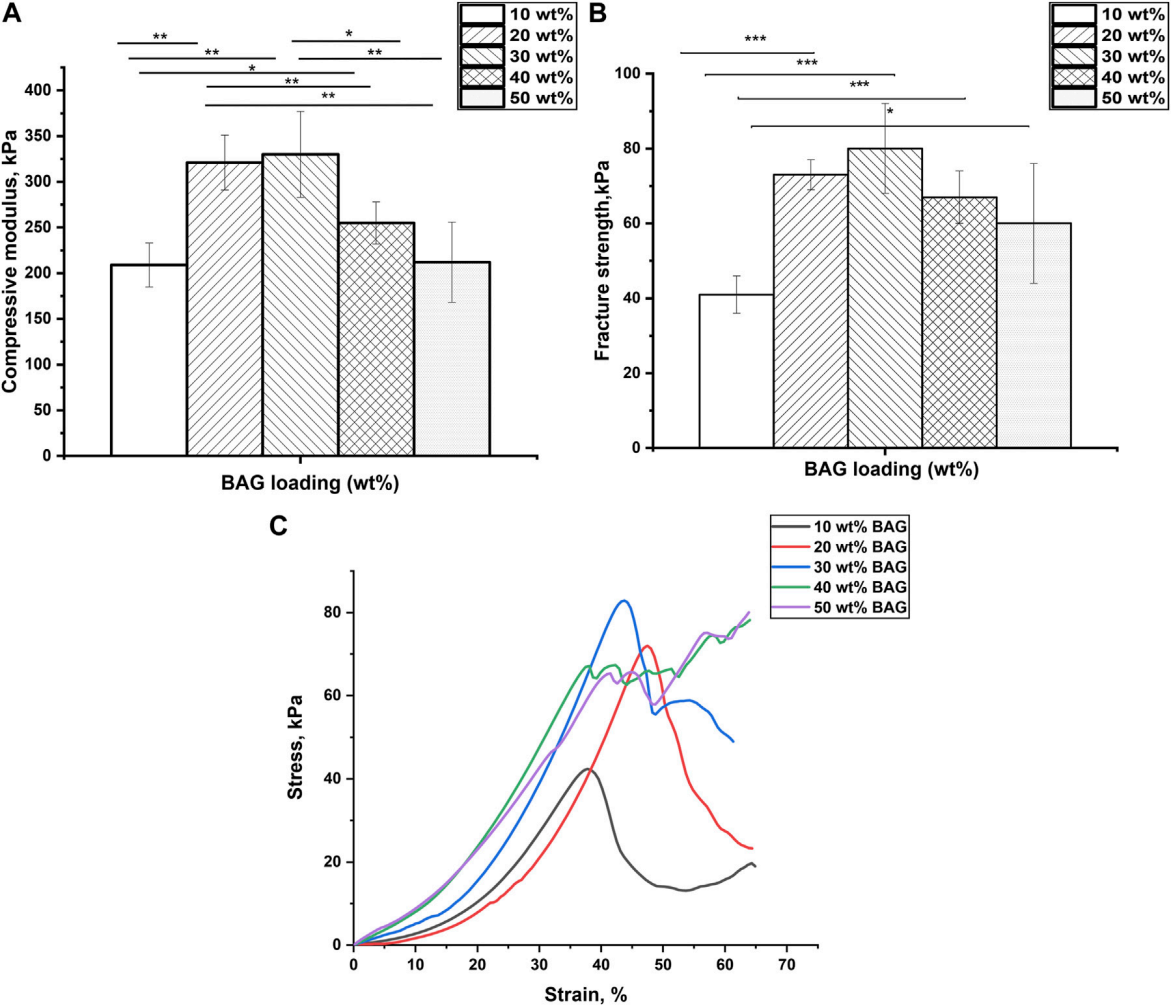
**(A)** Compressive modulus and **(B)** fracture strength of GG/BAG samples with ratio 90/10, 80/20, 70/30, 60/40, and 50/50 wt% at day 0. Data is presented as the mean ± standard deviation. A significant difference with *p* < 0.05 (*), *p* < 0.01 (**), and *p* < 0.001 (***). Significant differences can be observed from the GG/BAG 90/10 wt% samples to GG/BAG 80/20 wt% samples, to GG/BAG 70/30 wt% samples, and to GG/BAG 60/40 wt% samples; from GG/BAG 80/20 wt% samples to GG/BAG 60/40 wt% samples and to GG/BAG 50/50 wt% samples; from GG/BAG 70/30 wt% samples to GG/BAG 60/40 wt% samples and to GG/BAG 50/50 wt% samples. **(C)** Representative stress–strain curves of GG/BAG 90/10, 80/20, 70/30, 60/40, and 50/50 wt% at day 0.

To investigate the impact of BAG loading on the mechanical properties of GG/BAG composites, the mechanical properties of the samples were obtained by performing a compression test. The compressive modulus, fracture strength, and representative stress–strain curves are illustrated in Figures 3A–C. More detailed analysis of the stress–strain curves is presented in Supplementary Material S1.

Figures 3A,B show that GG/BAG 80/20 wt% and GG/BAG 70/30 wt% samples have the highest compressive modulus and fracture strength, i.e., 321 kPa and 330 kPa and 73 kPa and 80 kPa, respectively. The values from both GG/BAG 80/20 wt% and GG/BAG 70/30 wt% samples have no statistical difference (*p* = 0.76 for compressive modulus and *p* = 0.2 for fracture strength). Figure 3C exhibits examples of stress–strain curves, representative of each composite. The stress-strain curves reveal changes in compressive behavior above 30 wt%. Specifically, BAG loading <30 wt% exhibits a well-defined peak at maximum stress, whereas BAG loading >30 wt% results in a plateau at the maximum stress. This can be assigned to the large number of particles that undergo rearrangement within the gel matrix.

The increase in mechanical properties for the samples loaded with 10–30 wt% BAG can be explained by the addition of BAG microparticles, which help reinforce the mechanical properties of the composites. Since the BAG compressive modulus is higher than that of GG’s, the resulting compressive modulus of the composite will increase with an increase in BAG loading Callister and Rethwisch (2018). In addition, while an increase in BAG loading should improve the mechanical properties, here, the increase was only seen for loading up to 30 wt% BAG. The samples with 40 wt% and 50 wt% of BAG demonstrate a decline in mechanical properties (Figure 3A). The reduction in the mechanical properties can be ascribed to the decrease in the interfacial interactions between the polymer matrix and BAG microparticles (Feng et al., 2016). In addition, BAG particles in samples with 40 and 50 wt% of BAG are forming clusters by reaching a rigidity percolation point. Once the filler fraction exceeds the rigidity percolation point, the particles start to agglomerate leading to a decrease in interfacial cohesion, which leads to a decline in mechanical properties (Leuenberger and Ineichen, 1998; Verbeek, 2007). However, the mechanical properties of the composites are not only affected by the presence of BAG as a physical entity in the composite but also by interacting with the ions being released during the composite processing. As discussed previously in the work of Tang et al. (1995), the mechanical properties of these composites are affected by the formation of ionic bonding between Ca^2+^, Mg^2+^, K^+^, and Na^+^ cations and carboxylate anions of GG. It is therefore realistic to assume that potential crosslinking occurs between cations being released from the BAG into GG during the processing. Table 4 reports Ca^2+^ and Mg^2+^ ion concentrations in the solution released during the processing along with the number of crosslinking sites. As a result, the interaction site between GG and ions from the BAG occurs, which will constitute junction zones (crosslinks). For example, when all the possible crosslinks are built, the maximum number of complete junction zones is formed, and thus, the hydrogel network reaches its maximum strength (Tang et al., 1995). From the release study, and assuming preferential crosslinking between the GG and the divalent cations, the optimum BAG content to crosslink all junction zones has been estimated.

**TABLE 4:** Comparison between crosslinking sites and Ca^2+^ and Mg^2+^ released from the BAG.

GG/BAG wt%	90/10	80/20	70/30	60/40	50/50
Crosslinking sites	629	559	489	419	349
Possible crosslinks	314	279	244	209	174
Ca^2+^	64	117	288	508	674
Mg^2+^	13	24	58	101	138
Formed crosslinks	77	141	346	609	812
Crosslinking sites bonded with divalent ions	154	282	All	All	All
Non-crosslinked sites	475	277	None	None	None
Excess of ions in crosslinking	None	None	102	400	638

We excluded the number of monovalent ions because sucrose will undergo hydrolysis in the presence of organic sulfonic acid (HEPES) at acidic pH (pH = 6.45–6.50) and degrade to glucose and fructose. Glucose, fructose, and HEPES will form salts with Na^+^ and K^+^ (Buttersack et al., 2021).

Another factor negatively influencing the mechanical properties of samples 40 and 50 wt% BAG is the presence of an excessive amount of divalent ions, which creates competition for the formation of ionic bonds with the carboxyl groups in GG (Table 4), leading to weakened crosslinks in the junction zones and a decrease in mechanical properties (Tang et al., 1995). To conclude, BAG loading, distribution, and the number of crosslinking ions per crosslinking sites in the composite impact the material’s mechanical properties. The samples with a BAG loading of 10–30 wt% have a rising trend in mechanical properties, reaching a maximum in compressive modulus and fracture strength at 20 and 30 wt% of BAG in the sample. This could be explained by the rule of mixture (Callister and Rethwisch, 2018) and the rising number of divalent cations per available crosslinking sites in the system. The samples with a BAG loading of 40–50 wt% exhibit a decrease in mechanical properties due to the clustering of particles and competition between divalent ions per available crosslinking sites in the system and, thus, the presence of weaker crosslinks.

Previous studies (Tang et al., 1995; Verbeek, 2007; Douglas et al., 2014; Vuornos et al., 2019) have investigated the effect of divalent ionic crosslinking on the mechanical properties of GG. In our work, we focus on the impact of ion concentration in relation to the number of available crosslinking sites on these properties. For example, Douglas et al. reported on gellan gum gels (0.7% w/v) enriched with various bioglasses (1% w/v) with particle sizes from 30–100 nm to 5 µm. Apart from the cell/material interaction, the authors assess the impact of ions released from the glass on the mechanical properties of their composite, before and post immersion in SBF (Douglas et al., 2014). In this study, it is reported that the compressive modulus prior to immersion increases with the release of Ca^2+^ ions from the glass. Similar to the study by Douglas et al. (2014), we report an increase in the mechanical properties of the gel due to the release of divalent ions for the lower glass contents (up to 30 wt%). However, for the first time, we show that increasing glass loading from 30 wt% does not further improve the mechanical properties. Moreover, at higher loading, the mechanical properties of the gel decrease due to 1) competition between the divalent ions, for the available crosslinking sites (due to excess of divalent ions in the gel and 2) agglomeration of the glass particles leading to a decrease in the interfacial interaction between the matrix and the BAG particles.

### 3.2 *In vitro* dissolution studies of GG/BAG samples

#### 3.2.1 *In vitro* dissolution in PBS

##### 3.2.1.1 *In vitro* dissolution in PBS and changes in mass and pH

**FIGURE 4:**
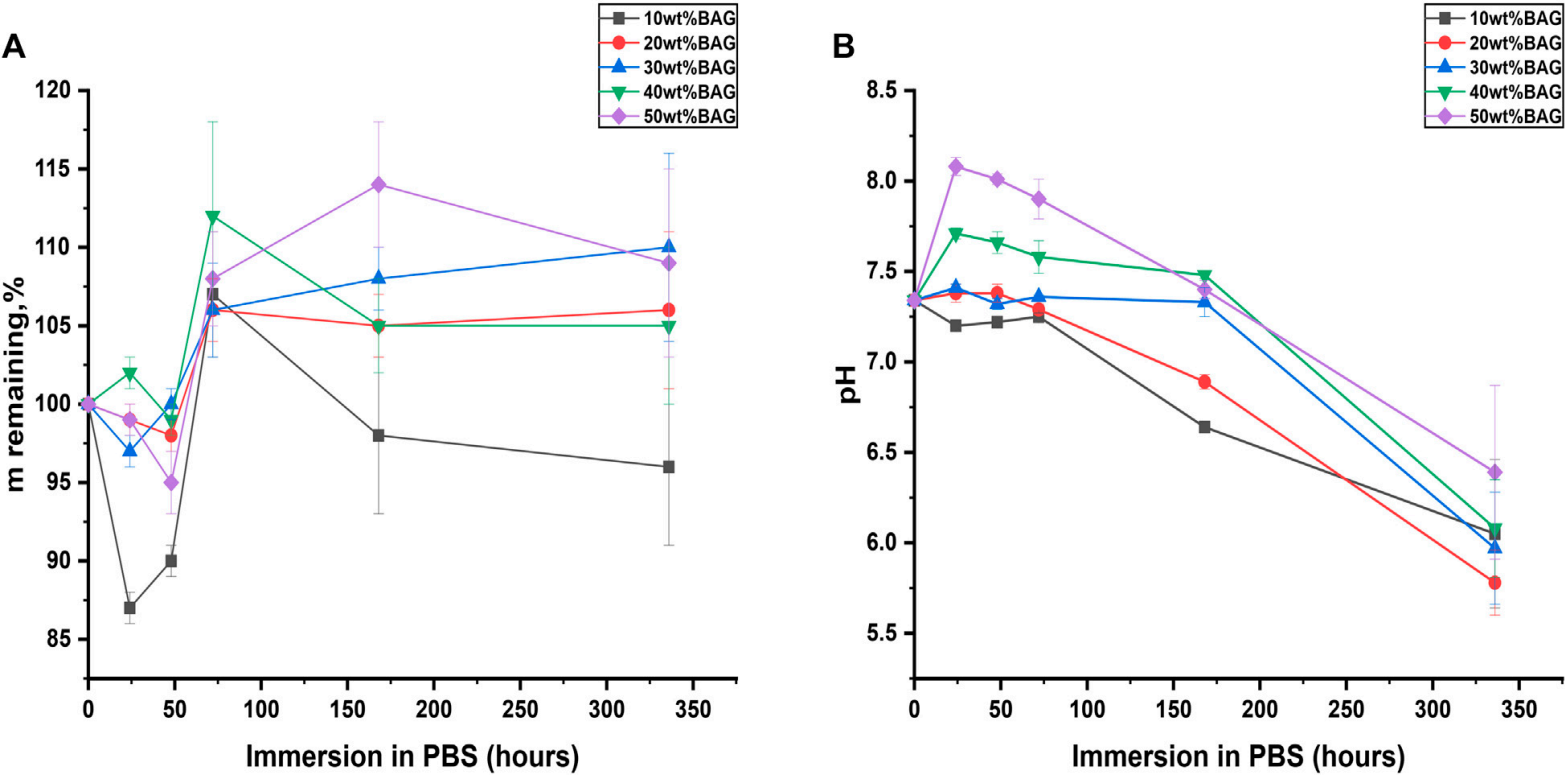
**(A)** Mass remaining and **(B)** changes in pH upon immersion of the GG/BAG composites.

**FIGURE 5:**
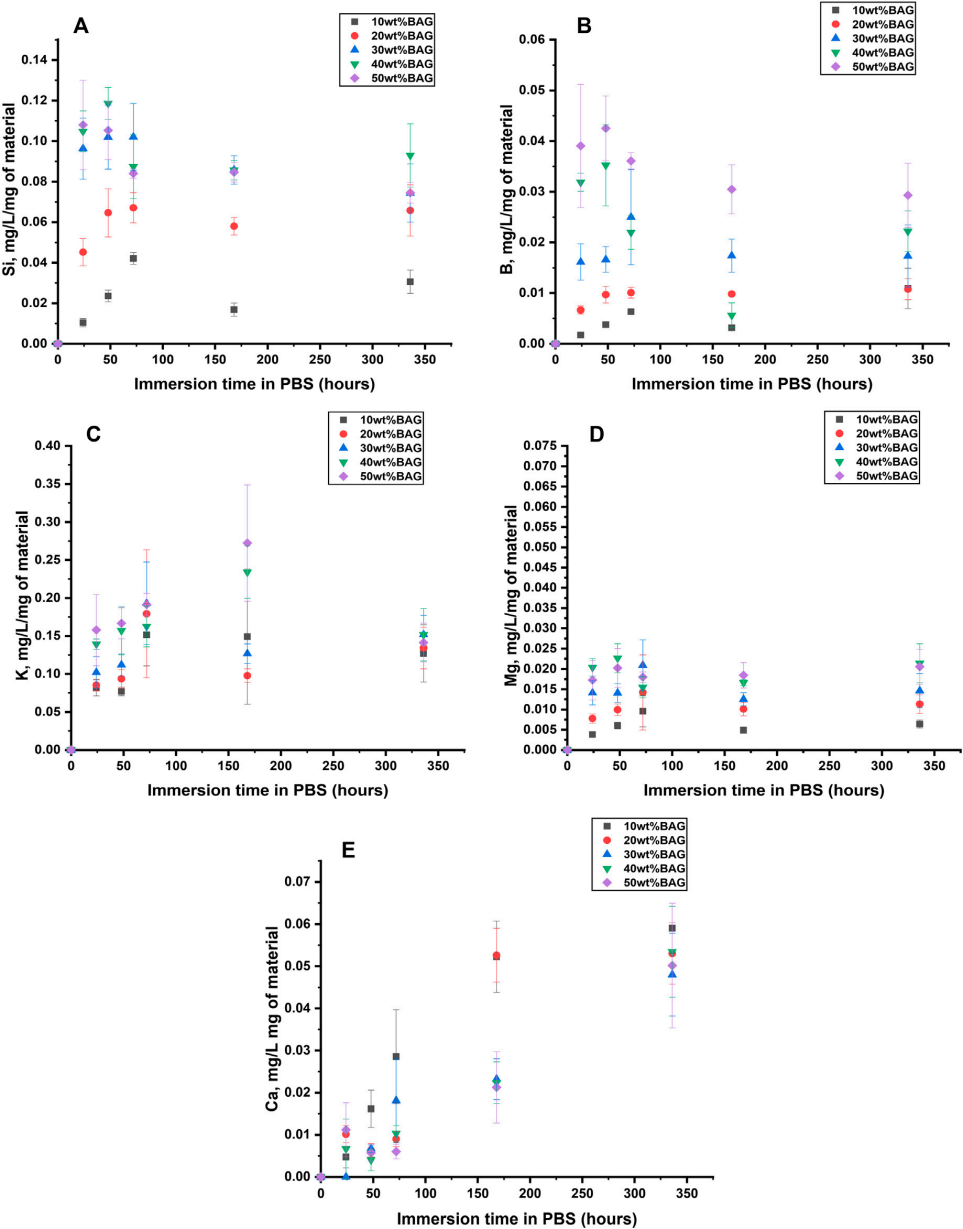
**(A)** Si, **(B)** B, **(C)** K, **(D)** Mg, and **(E)** Ca Ion concentration in the upon immersion of the GG/BAG composites in PBS. The concentrations are normalized to the sample mass.

Here, we evaluated the impact of BAG on the hydrolytic degradation of composites. The mass and pH changes as a function of immersion time are presented in Figures 4A, B and Supplementary Material S3. As shown in Figure 4A, samples with 10 wt% of BAG had a reduction of mass to 87 ± 1% on day 1 and further exhibited an increase in the mass to 96% ± 5% on day 14. Samples with 20, 30, 40, and 50 wt% of BAG had non-significant changes in their mass on day 1. However, the masses of all the samples witnessed a mass increase up to 106%–112% on day 3 and stabilized to 106%–110% on day 14 of immersion in PBS. The changes in the samples’ masses can be attributed to the competition between 1) the hydrolytic degradation of GG with the appearance of D-glucuronic acid as a product of GG degradation, 2) crosslinking of the GG chains by the ions released from the BAG particles, and 3) the precipitation of a Ca–P layer due to the saturation of the solution by ions released from the BAG particles. One can assume from the results in Figure 4A that the higher the concentration of BAG in the GG, the lower the effect of hydrolytic degradation on the samples. This can be attributed to the interactions between the carboxylate anions on the GG and divalent cations from the BAG, decreasing the available hydrogen bond donors and acceptors, thus limiting the interaction with water. The pH changes for each sample between day 1 and day 14 of immersion are presented in Figure 4B. From the results, we can see that during the first day, the samples containing higher amounts of BAG (20, 30, 40, and 50 wt%) had the most significant changes in pH starting at 7.34 and reaching up to 7.41–8.08. Successively, these samples exhibit a significant drop in pH to 5.78–6.39. The initial increase in pH could be explained by the release of cations (Na^+^ and K^+^) from BAG in the solution and subsequent ion exchange with H^+^ (Figure 5). The decrease in pH might be associated with the degradation of GG. Samples with 10 wt% of BAG show a decrease in pH already at day 1 (from 7.34 to 6.05 ± 0.41). This can be assigned to the low content in glass and faster degradation of GG.

##### 3.2.1.2 Ion release from BAG as a function of immersion in PBS

To identify the impact of ions released from BAG on the behavior of the composites in PBS, we measured ion concentration in the solution as a function of immersion time (Figure 5). The ion concentration was normalized to the mass of the sample. According to the dissolution mechanism of borosilicate BAG, Na^+^ and K^+^ released fast on the first day (Houaoui et al., 2021). K^+^ release was in agreement with the rise in pH within day 1. Si^4+^ and B^3+^ increase during the first 3 days and then stabilize until day 14. This can be assigned to the fast release of the soluble silicate and repolymerization of the insoluble silica in the glass structure, thus forming a SiO_2_-rich gel at the glass surface and reducing the release of other ions. Mg^2+^ rises during the first 3 days and stabilizes after that. It is hypothesized that Ca^2+^ and P^5+^ will precipitate in 7 days. Our experimental results indicate that samples with higher BAG loading exhibit lower concentrations of Ca^2+^ in the immersion solution compared to those with lower glass loading. This suggests faster precipitation of a reactive layer for the composites with high glass content. The high concentration in Na^+^ and P^5+^ in PBS prevents the accurate quantification of these ions post immersion.

##### 3.2.1.3 Swelling ratio and mechanical properties post-immersion

**FIGURE 6:**
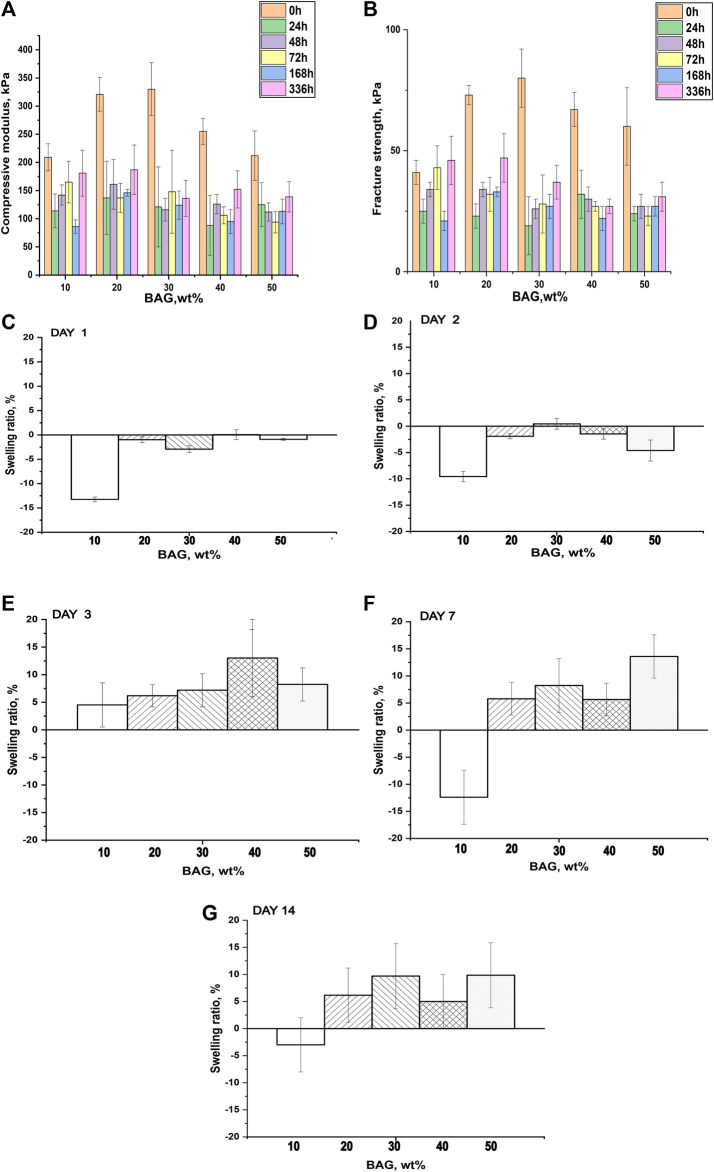
**(A)** Compressive modulus, **(B)** fracture strength, and **(C–G)** swelling ratio of GG/BAG samples after immersion in PBS at 1, 2, 3, 7, and 14 days.

To assess how BAG impacts the swelling of the samples, we measured the swelling ratio of materials post-immersion in PBS. The swelling ratio was compared with the mechanical properties of our GG/BAG composites (Figure 6, Supplementary Material S4). All samples underwent de-swelling at up to 2 days of immersion and started to swell on day 3. The mechanical properties of composites significantly decreased after day 1 of immersion in PBS. A drop in mechanical properties (Figures 6A, B) occurred due to increased porosity and subsequently decreased the interfacial interconnection between the gel and BAG and in crosslinking density. The lower the BAG content, and the higher the number of non-ionically bonded carboxylate anions of GG in the GG/BAG composite, the higher the interaction of these free carboxylate anions with water molecules (Figures 6C–G) during immersion in aqueous media.

#### 3.2.2 Enzymatic degradation

##### 3.2.2.1 Enzymatic degradation and changes in mass and pH

**FIGURE 7:**
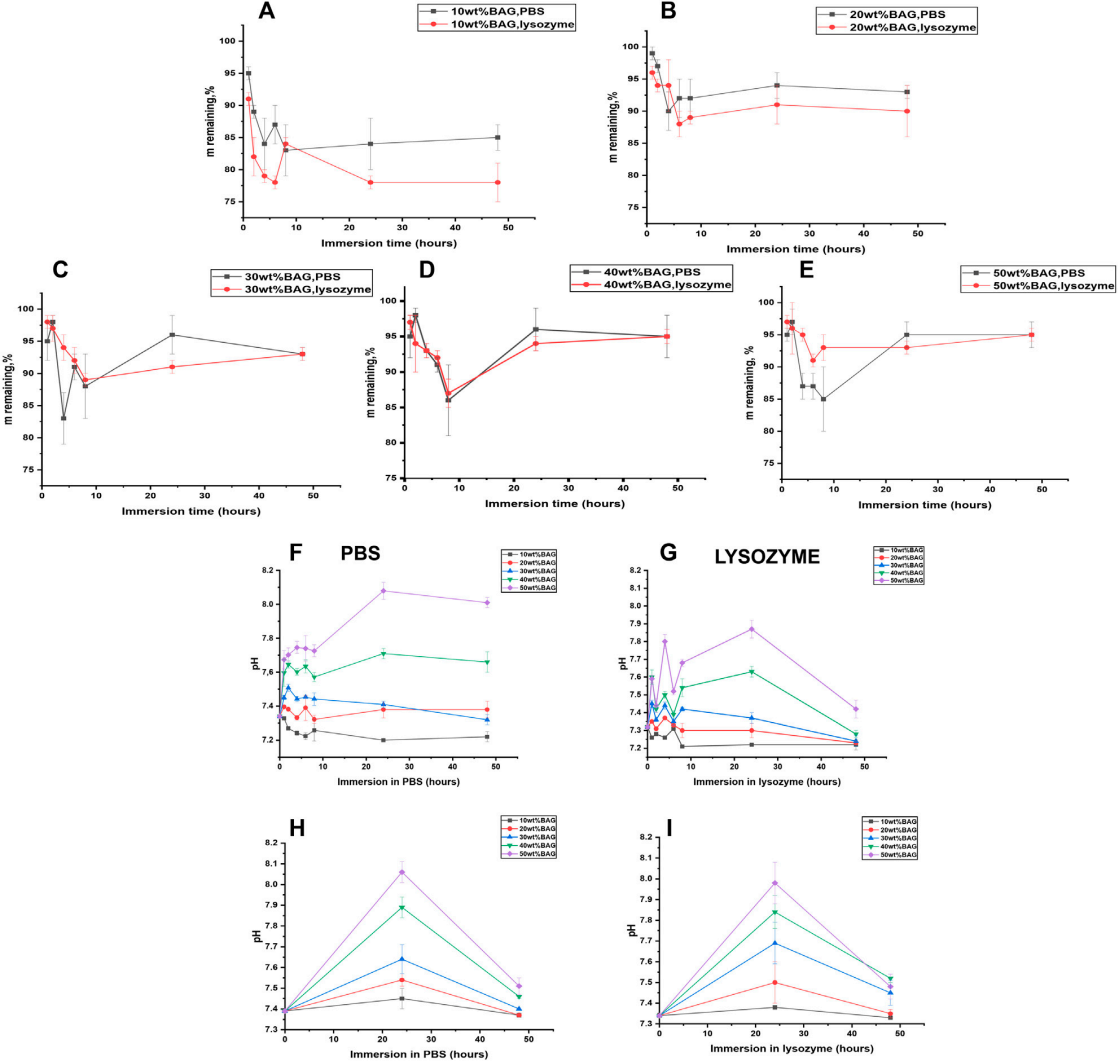
GG/BAG samples mass remaining upon immersion in PBS and lysozyme: **(A)** 90/10 wt%, **(B)** 80/20 wt%, **(C)** 70/30 wt%, **(D)** 60/40 wt%, and **(E)** 50/50 wt%. Changes in pH for GG/BAG samples immersed in **(F)** PBS and **(G)** lysozyme; changes in pH for 10–50 wt% of BAG without GG immersed in **(H)** PBS and **(I)** lysozyme.

We immersed the samples in lysozyme-containing PBS to assess the impact of BAG addition in GG on enzymatic degradation. Lysozyme-free PBS was used as a control. Changes in mass are illustrated in Figure 7A–E. The pH changes for all GG/BAG samples immersed for 1–48 h are presented in Figures 7F–G, and pH changes for BAG alone at 24 and 48 h are illustrated in Figures 7H–I.

As can be seen from Figure 7A, enzymatic degradation by lysozyme reduces the mass of GG/BAG 90/10 wt% samples to 78 ± 1% compared to 85 ± 1% mass for the same samples immersed in PBS for 48 h. This can be explained by the breakage of ether bonds connecting the structural backbone of polysaccharides by lysozyme (Xu et al., 2018). Other GG/BAG (80/20, 70/30, 60/40, and 50/50 wt%) samples experience mass loss after immersion in both lysozyme (to 90–95%) and PBS (to 93–95%). It is interesting to note that there are no changes in the mass loss for GG/BAG 70/30, 60/40, and 50/50 wt% samples immersed in lysozyme compared to the same samples immersed in PBS, as illustrated in Figures 7C–E. This can be explained by a higher degree of crosslinking that prevents lysozyme from attacking ether bonds.

The pH increases for 60/40 and 50/50 wt% GG/BAG samples over 24 h from 7.34 to 7.71–8.08 (PBS) and from 7.32 to 7.63–7.87 (lysozyme) (Figures 7F,G). Then, the pH declines for 60/40 and 50/50 wt% GG/BAG samples from 7.71–8.08 to 7.66–8.01. On the other hand, the pH for 90/10, 80/20, and 70/30 wt% did not show any noticeable changes within all 48 h: for 90/10 wt% it changes from 7.34 ± 0.01 to 7.22 ± 0.02 (PBS), and from 7.32 to 7.22 ± 0.02 (lysozyme), for 80/20 wt%—from 7.34 to 7.38 ± 0.01 (PBS), and from 7.32 to 7.23 ± 0.04, for 70/30 wt%—from 7.34 to 7.32 ± 0.01 (PBS), and from 7.32 to 7.24 ± 0.01 (lysozyme). This can be explained by the higher amount of alkaline ions and subsequently high release in 60/40 and 50/50 wt% GG/BAG samples for the first 24 h in PBS and lysozyme and then stabilization from 24 to 48 h in the PBS. A decline in pH for 60/40 and 50/50 wt% GG/BAG at lysozyme occurred due to minor formation of D-glucuronic acid.

To conclude, the effect of enzymatic attack is reduced due to the higher crosslinking density, as can be seen from Figures 7A–E. The higher the BAG amount in the sample, the higher the remaining mass. The pH increases for GG/BAG and BAG samples (Figures 7F–I) in 24 h occurred due to dominant glass dissolution and release of alkaline ions.

##### 3.2.2.2 Ion release from BAG as a function of immersion in lysozyme

**FIGURE 8:**
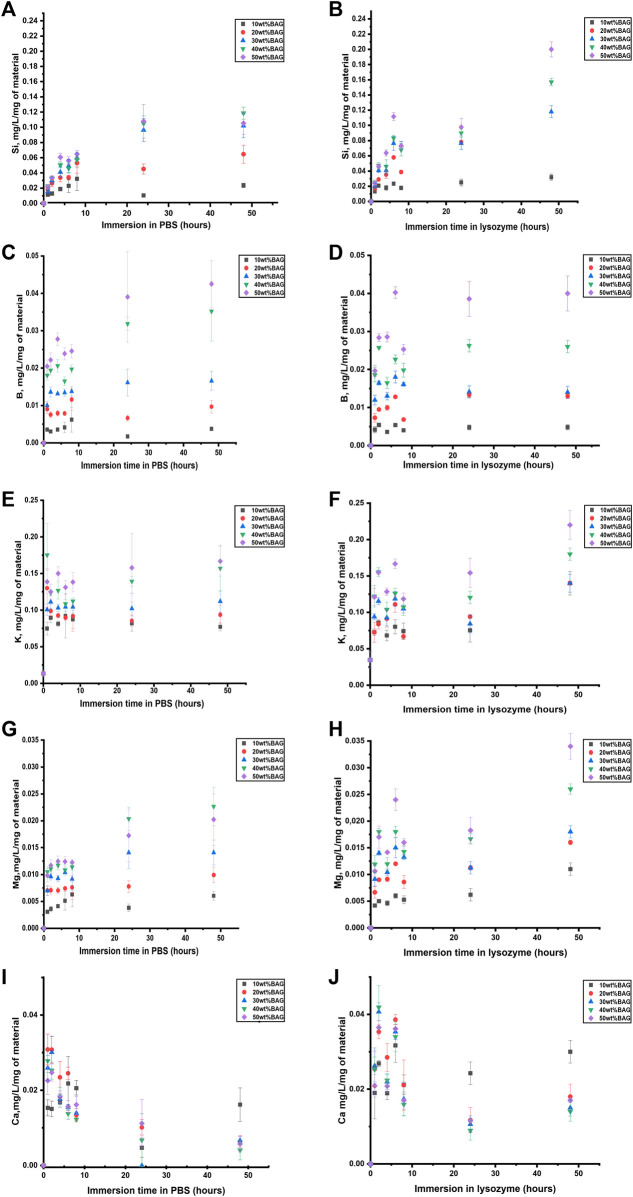
**(A, B)** Si, **(C, D)** B, **(E, F)** K, **(G, H)** Mg, and **(I, J)** Ca ion concentration upon immersion in **(A, C, E, G, I)** PBS and **(B, D, F, H, J)** lysozyme, as a function of time. The concentrations are normalized to the sample mass.

To identify the impact of ions released from BAG on the behavior of the composites in lysozyme, we measured the ion concentration in the solution at the same time points as remaining mass and pH. The ion concentration in lysozyme and PBS was assessed by ICP-OES. The ion release from the GG/BAG samples in lysozyme-containing PBS and PBS only, as a function of immersion time, is presented in Figures 8A–J.

**FIGURE 9:**
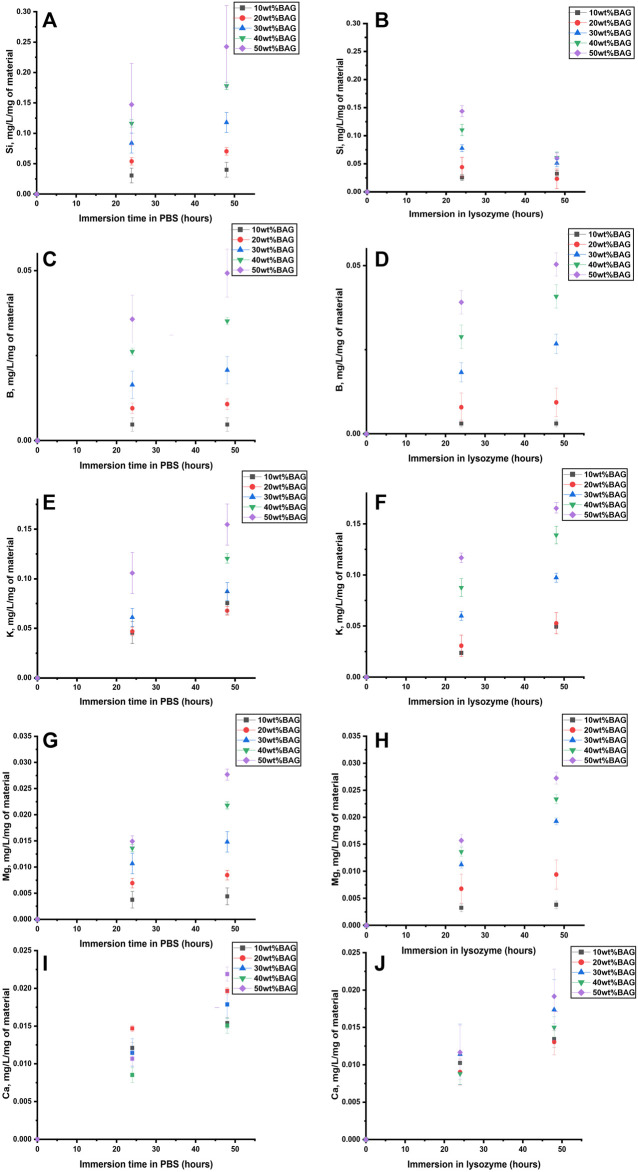
**(A, B)** Si, **(C, D)** B, **(E, F)** K, **(G, H)** Mg, and **(I, J)** Ca ion concentration upon immersion of BAG alone in **(A, C, E, G, I)** PBS and **(B, D, F, H, J)** lysozyme as a function of time. The concentrations are normalized to the sample mass.

As can be seen in Figures 8A,B and Figures 9A,B, Si^4+^ ion release from GG/BAG samples in PBS increased at 24 h by 0.1 mg/L/mg of material and stabilizes, while in lysozyme, it doubled (from 0.1 to 0.2 mg/L/mg of material) from 24 to 48 h. Si^4+^ ion release from BAG alone, in PBS, increased from 0.15 to 0.24 mg/L/mg of material. As for boron release (Figures 8C,D and Figures 9C,D), there is a significant difference in the release of B^5+^ in lysozyme for GG/BAG samples compared to BAG alone, while the release from GG/BAG in PBS is higher than that from BAG alone, Figures 8E–J and Figures 9E–J show that there are no significant differences in the release of K^+^, Mg^2+^, and Ca^2+^ in PBS and lysozyme/PBS for both GG/BAG samples and BAG alone.

It can be observed that the release of alkaline ions from the GG/BAG and BAG (Figures 8E, 9E) results in changes in pH (Figures 7F,G) during immersion in both PBS and lysozyme/PBS, and pH increases at 24 h and decreases from 24 to 48 h’ time points.

K^+^ and Mg^2+^ in GG/BAG increase at 48 h in lysozyme, while the pH remains stable in PBS. This can be observed especially in 80/20, 70/30, 60/40, and 50/50 wt% GG/BAG (Figures 7F, 8E) samples. The samples with 20, 30, 40, and 50 wt% of BAG show an increase in K^+^, with the pH rising only for the samples with 30, 40, and 50 wt% (Figures 7G, 8F). The appearance of D-glucuronic acid as a GG degradation product negates the pH change. For samples with 10 wt% of BAG in PBS and for samples with 10 wt% and 20 wt% of BAG in lysozyme, the pH is more stable and the weight loss is higher than for samples with higher BAG loading. K^+^ and Mg^2+^ in GG/BAG increased at 48 h in lysozyme, while it remained stable in PBS (Figures 8E–H; Figures 9E–H). This can be attributed to the weaker bonds formed between COO^−^ and K^+^/Mg^2+^, when compared to Ca^2+^, leading to a release of the cations under enzymatic degradation (Morris et al., 2012).

In addition, Si^4+^ release in GG/BAG samples increased from 24 to 48 h in GG/BAG samples in lysozyme, while it remained stable for the same samples in PBS (Figures 8A,B). At the same time, Si^4+^ release in BAG increased in PBS and declined in lysozyme in 48 h (Figures 9A,B). This can be evidence of the higher degradation of GG/BAG in lysozyme and the inability of Si^4+^ to polymerize on the GG/BAG sample in 48 h.

The stability of GG gels and GG-based composites have been extensively studied (Bacelar et al., 2016; Xu et al., 2018; Bonfacio et al., 2020). Xu et al., for example, examined the effects of the different crosslinking mechanisms on four high-molecular weight (HMGG) and 10 low-molecular weight (LMGG) GG compositions and their degradation profiles upon immersion in 0.5 mg/ml lysozyme for up to 16 days. In our study, the samples were immersed in 3.0 mg/ml lysozyme/PBS solution for up to 2 days. Xu reported that after 16 days, the samples degraded to 20% of their initial weight due to the breakage of ether bonds connecting the structural body of GG and lysozyme (Xu et al., 2018). The mass of HMGG samples in Xu’s work decreased after 2 days to 70–75% mass remaining in lysozyme/PBS, while in our work, 90/10 wt% GG/BAG samples decreased to 78% mass remaining in lysozyme/PBS, 80/20 wt% GG/BAG samples to 90% mass remaining, and 70/30, 60/40, and 50/50 wt% GG/BAG samples to 93–95% mass remaining, having the same decrease in mass as compared to PBS. Here, we demonstrated that increasing the degree of crosslinking by divalent ions released from BAG increases the resistance to enzymatic degradation, even at higher lysozyme concentration when compared to previous studies (Xu et al., 2018).

#### 3.2.3 Bioactivity in SBF

The samples were immersed in SBF (Kokubo, 1991) to study their bioactivity. pH measurements, ICP-OES analysis, X-ray fluorescence analysis, and microcomputed tomography (μCT) were performed to assess the dissolution of BAG and the ability of the composite to precipitate an HA layer (Kamistos and Chryssikos, 1991).

##### 3.2.3.1 *In vitro* dissolution in SBF, pH changes, and ion release from BAG as a function of time during immersion in SBF

**FIGURE 10:**
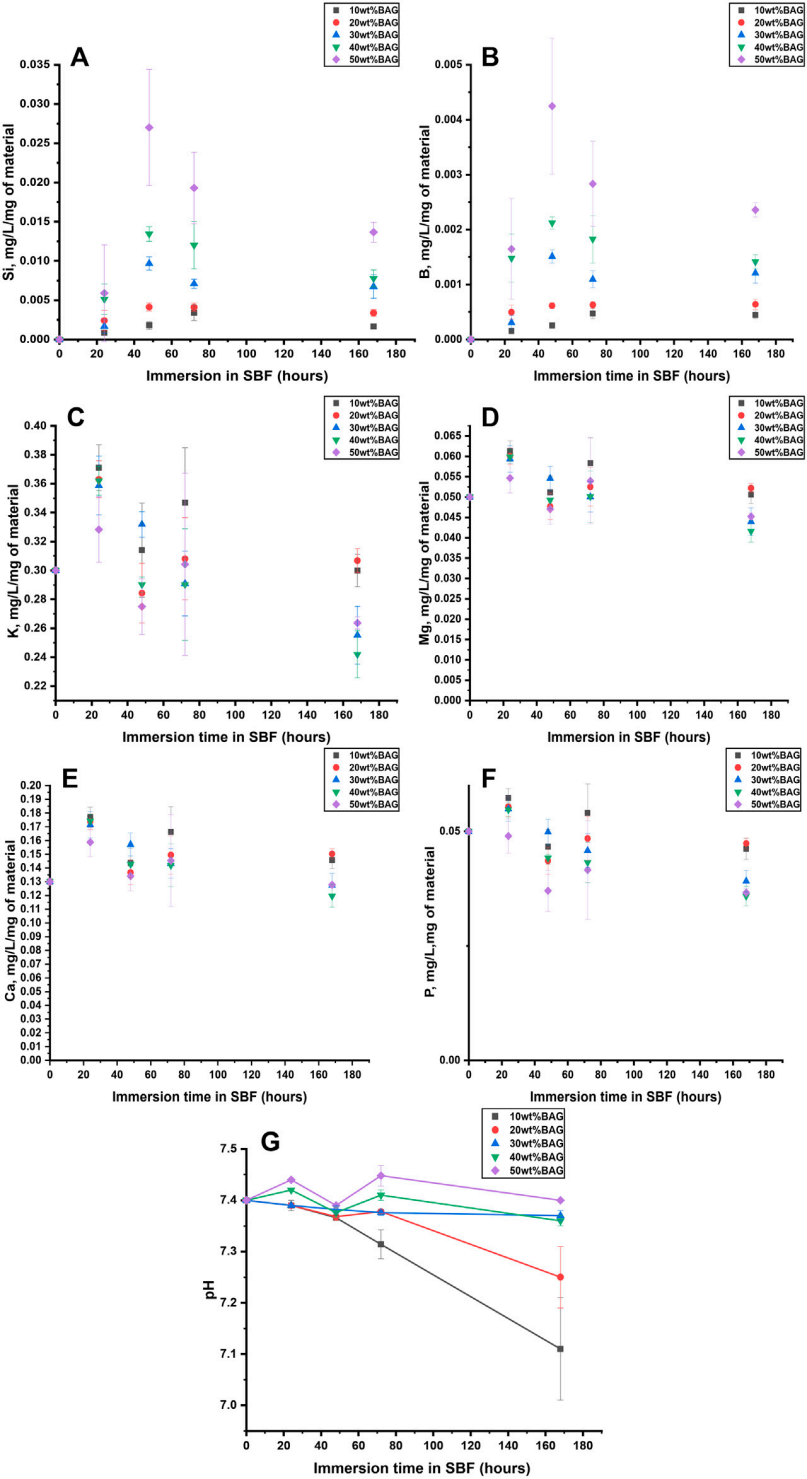
**(A)** Si, **(B)** B, **(C)** K, **(D)** Mg, **(E)** Ca, and **(F)** P ion concentration upon immersion of the GG/BAG samples in SBF. The concentrations are normalized to the sample mass; **(G)** pH changes of the SBF as a function of immersion time.

The changes in ion release and pH during immersion in SBF are presented in Figures 10A–G.

As can be seen from Figure 10G, the pH in the solution at day 0 is 7.40. The ions in the solution were quantified by ICP-OES and normalized to the mass of the samples. The pH of the solution for samples with 30, 40, and 50 wt% of BAG remains within 7.36–7.45 for 7 days of immersion. The pH of samples with 10 and 20 wt% decreases to 7.31–7.38 on day 3, with a sharp decline to 7.11–7.25 from day 3 to day 7. The samples with 40 and 50 wt% of BAG increase in pH on day 1 due to a sharp increase of Na^+^ and K^+^, while for other samples the pH decreased due to dominant hydrolytic degradation of GG with the formation of D-glucuronic acid. Si^4+^ and B^5+^ release increases at day 2 and decreases from 2 to 7 days, most likely due to the polymerization of a Si-rich layer at surface of the glass particles (Jones, 2013). The Ca^2+^ and P^5+^ concentrations fluctuate and then decrease for samples with 40 and 50 wt% of BAG from day 3 to day 7. A decrease of Ca^2+^ and P^5+^ indicates precipitation of the Ca–P layer for samples with 30, 40, and 50 wt%. As for other ions, Mg^2+^ release shows a similar trend as P^5+^. However, K^+^ has a similar ion release as Ca^2+^, which can be addressed by the incorporation of Mg^2+^ and K^+^ in the Ca–P reactive layer.

The 90/10 and 80/20 wt% GG/BAG samples immersed in PBS (Figures 4B, 7F), SBF (Figure 10G), and lysozyme/PBS (Figure 7G) show a significant decline in pH in 7 days in both PBS and SBF due to the degradation of GG. Samples with 30, 40, and 50 wt% do not change drastically in their pH because the BAG in these samples inhibits hydrolytic degradation in 7 days. In 48 h (2 days), the pH of the 40 and 50 wt% samples increases, while it declines for 10, 20, and 30 wt% samples in both lysozyme and SBF. This occurred because of the higher degradation in the hydrolytic and enzymatic conditions of the samples immersed in SBF and lysozyme/PBS, compared to hydrolytic degradation occurring only in PBS. BAG in samples with 40 and 50 wt% inhibits both hydrolytic and enzymatic degradation on day 2 in all three media.

Also, Figures 5E, 10E show Ca^2+^ release in both PBS and SBF. In both solutions, samples with the least BAG loading (10 wt%) had the highest Ca^2+^ release, which can be explained by the possible precipitation of Ca^2+^ on the samples with 20, 30, 40, and 50 wt% of BAG.

Houaoui et al. (2021) analyzed the *in vitro* dissolution of hybrid scaffolds based on gelatin and the same BAG 13-93B20, covalently crosslinked using GPTMS (70/30 wt%) in SBF for up to 14 days. In our research, we tested the samples in SBF for up to 7 days, and the samples with the highest wt% of BAG show a decrease in Ca^2+^ on day 7, indicating the precipitation of hydroxyapatite.

During *in vitro* dissolution in SBF, in Houaoui’s work, Ca^2+^ started to decrease after 7 days of immersion, while in our work, Ca^2+^ decreased between day 3 and day 7, which is evidence of precipitation of the Ca–P layer on the top of the sample. Moreover, samples with 30, 40, and 50 wt% BAG loading have a higher decline in Ca^2+^ release. Thus, the higher the BAG loading, the higher the Ca–P layer formation.

##### 3.2.3.2 Dissolution and reaction of BAG microparticles in SBF

**FIGURE 11:**
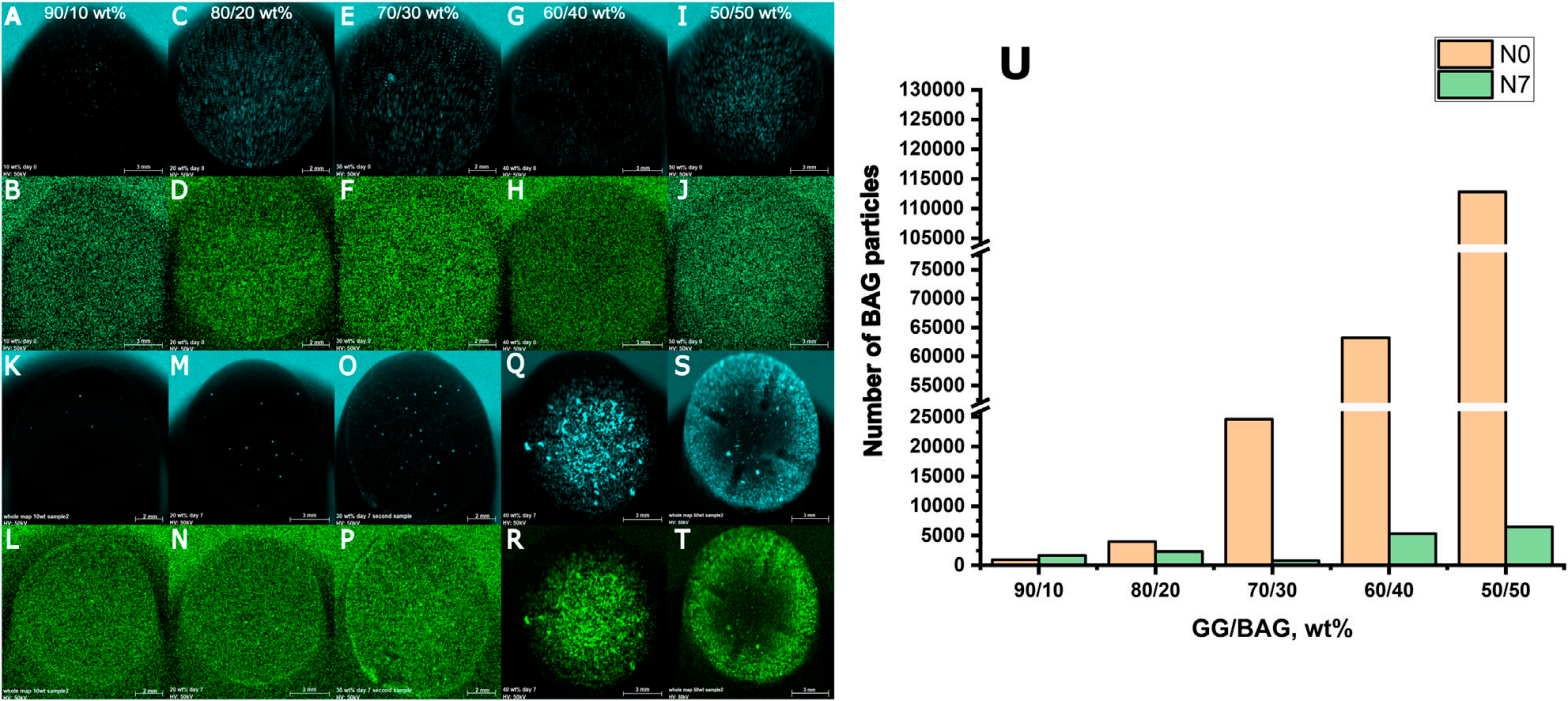
XRF images of the surfaces of GG/BAG samples at day 0 (first and second rows) and day 7 (third and fourth rows) of immersion in SBF: **(A, K)** Ca and **(B, L)** P in GG/BAG 90/10 wt% samples; **(C, M)** Ca and **(D, N)** P in GG/BAG 80/20 wt% samples; **(E, O)** Ca and **(F, P)** P in GG/BAG 70/30 wt%; **(G, Q)** Ca and **(H, R)** P in GG/BAG 60/40 wt% samples; **(I, S)** Ca and **(J, T)** P in GG/BAG 50/50 wt% samples; and **(U)** number of BAG particles in the GG/BAG samples before (N0) and 7 days post immersion (N7) in SBF.

XRF images of the composite surface and a number of BAG particles before (N0) and after 7 days of immersion in SBF (N7) are presented in Figure 11 (A–U).

The results from X-ray fluorescence analysis (Figures 11A–T) show evidence of the formation of the Ca–P reactive layer. This can be explained as the precipitation of the Ca–P layer. Moreover, using X-ray microcomputed tomography (Figure 1), analysis of the distribution of the particles in the sample before and post immersion was quantified and is presented in Table 3, Supplementary Material S2 (pixel size of 5.64 µm). Post-immersion, μCT images (Figure 1) show that particles are dispersed irregularly on the top of the sample containing 40 wt% BAG, and the distribution of particles on other samples is not visible. The number of particles 7 days post immersion in SBF decreased for all composites, except for the samples with 10 wt% of BAG. The decrease in particle number is expected due to the significant dissolution of the glass occurring during immersion. The increase in the number of particles for the composite containing 10 wt% of BAG can be assigned to either inhomogeneity across the sample and/or the low number of particles making the quantification highly inaccurate.

As shown by Douglas et al. (2014) and Houaoui et al. (2021), the incorporation of bioactive glasses led to the precipitation of a Ca–P layer, most likely hydroxyapatite. However, the low bioactive glass content in GG suffers from early glass dissolution during sample preparation and, therefore, slow and sporadic precipitation of HA. Glass content above 30 wt% leads to a rapid precipitation of hydroxyapatite, covering the entire surface of the composite.

## 4 Conclusion

Here, we demonstrate that the dissolution of BAG during the processing of GG/BAG composites leads to the release of cations that are able to interact with the GG chains. The cations were found to interact with the carboxylate anions of the GG. Such an increase in the crosslinking density led to an increase in the fracture strength and modulus, along with an increased hydrolytic resistance both with and without lysozyme.

While the hydrolytic resistance was improved, with increasing glass content, the mechanical properties exhibit a maximum at 30 wt% loading. This maximum was assigned to the number of cations in the gel exceeding the number of available crosslinking sites and to the agglomeration of the particles, leading to a decrease in the interface between the glass particles and the matrix.

The presence of BAG particles in the composite was also found to impact the swelling of the materials. Indeed, the higher the glass content, the higher the swelling post immersion. This was clearly attributable to the dissolution of the glass particles, leading to increased porosity over the course of the dissolution test.

Finally, it was evidenced by XRF analysis that a low content of BAG did not lead to significant precipitation of HA. The HA reactive layer was only found to precipitate at the surface of composites with 30 wt% or more of BAG.

These results provide a different understanding of the interaction between BAG and GG, along with critical data on the impact of early glass dissolution, during processing, on the overall composite properties. It is also shown that upon understanding the interaction between BAG and GG, one can tailor the composite composition to optimize the mechanical properties while guaranteeing the precipitation of HA upon immersion, seen as the first sign of bioactivity.

During *in vitro* dissolution in lysozyme, degradation of the samples with 30, 40, and 50 wt% of BAG is expressively reduced (Figures 7C–E). The enzymatic attack decreased with the increased number of crosslinks. Indeed, when measuring the remaining mass post immersion in lysozyme and PBS, it was demonstrated that BAG/GG 70/30, 60/40, and 50/50 wt% samples have higher remaining masses (Figures 7C–E) than samples with a smaller amount of the BAG (GG/BAG 90/10 and 80/20 wt%) (Figures 7A,B). To conclude, we evaluated the impact of chemical interactions between GG and BAG at five different weight ratios on crosslinking, mechanical properties, and *in vitro* dissolution. It has been found that the ratio between crosslinking ions from the BAG, crosslinking sites, and the number of crosslinks impact the mechanical properties and the *in vitro* degradation of GG/BAG composites. The 80/20 and 70/30 wt% GG/BAG samples have the highest mechanical properties, and both enzymatic and hydrolytic degradation is reduced in 70/30, 60/40, and 50/50 wt% GG/BAG samples. Hydroxyapatite is precipitated on 60/40 and 50/50 wt% GG/BAG samples upon immersion in SBF, which is an indication of bioactivity. After all the experiments, the samples with 30, 40, and 50 wt% BAG will be used for printability and biocompatibility.

## Data Availability

The original contributions presented in the study are included in the article/Supplementary Material; further inquiries can be directed to the corresponding author.

## Supplementary material

Supplementary Material S1
**(A–E)** Representative stress-strain curves of GG/BAG composites (compression testing).

Supplementary Material S2 Comparison of theoretical and measured (µCT) BAG distribution in composites.

Supplementary Material S3 Mass remaining and pH during *in vitro* studies.

Supplementary Material S4 Swelling ratio vs mechanical properties post-immersion in PBS.

Supplementary Figure S1 EM images of GG/BAG samples at day 0 (first row) and EDS mapping of Si showing the BAG distribution in the same areas (second row). Samples shown in the images are: **(A, B)** GG/BAG 80/20 wt%, **(C, D)** GG/BAG 70/30 wt%, (E, F) GG/BAG 60/40 wt%, and (G, H) GG/BAG 50/50 wt%.

Supplementary Figure S2 SEM image of BAG size and local distribution in GG/BAG samples.

Supplementary Figure S3 XRD patterns of GG powder, BAG powder, and GG/BAG samples (arbitrary units), peaks of interest are marked with and .

## Abbreviations

BAG, bioactive glass; GG, gellan gum; HEPES, 4-(2-Hydroxyethyl)piperazine-1-ethanesulfonic acid; ICP—OES—Inductively Coupled Plasma-Optical Emission Spectroscopy; PBS, phosphate-buffered saline; SBF, simulated body fluid; and TRIS, Tris (hydroxymethyl)aminomethane.
