# Supplementary material for: Chemical interactions in composites of gellan gum and bioactive glass: self-crosslinking and in vitro dissolution
Source: Front Chem. 2023 May 12;11:1133374. doi: 10.3389/fchem.2023.1133374 (PMC10213777; doi:10.3389/fchem.2023.1133374)
Supplement: Supplementary file 1 [file DataSheet4.docx]

**Supplementary material S4**

| **Immersion time in PBS** | **Day 0** | **Day 1** | **Day 2** | **Day 3** | **Day 7** | **Day 14** |
| --- | --- | --- | --- | --- | --- | --- |
| **GG/BAG 90/10 wt%** | | | | | | |
| **Swelling ratio, %** |  | **-13.3±0.5** | **-9.6±1.0** | **4.5±4.0** | **12.4±5.0** | **-3.0±5.0** |
| **Compressive modulus, kPa** | **209±24** | **114.0±30.0** | **142.0±18.0** | **165.0±37.0** | **86.0±12.0** | **187.0±44.0** |
| **GG/BAG 80/20 wt %** | | | | | | |
| **Swelling ratio, %** |  | **-1.0±0.6** | **-1.9±0.4** | **6.2±2.0** | **5.78±3.0** | **6.2±5.0** |
| **Compressive modulus, kPa** | **321±30** | **137.0±65.0** | **161.0±44.0** | **137.0±26.0** | **146.0±6.0** | **187.0±44.0** |
| **GG/BAG 70/30 wt %** | | | | | | |
| **Swelling ratio. %** |  | **-2.9±0.7** | **0.4±1.0** | **7.2±3.0** | **8.2±5.0** | **9.7±6.0** |
| **Compressive modulus, kPa** | **330±47** | **121.0±71.0** | **116.0±20.0** | **148.0±74.0** | **124.0±25.0** | **136.0±32.0** |
| **GG/BAG 60/40 wt %** | | | | | | |
| **Swelling ratio, %** |  | **0.1±1.0** | **-1.5±1.0** | **13.0±7.0** | **5.7±3.0** | **5.0±5.0** |
| **Compressive modulus, kPa** | **255±23** | **88.0±53.0** | **126.0±17.0** | **106.0±15.0** | **95.0±21.0** | **152.0±33.0** |
| **GG/BAG 50/50 wt %** | | | | | | |
| **Swelling ratio. %** |  | **-0.9±0.2** | **-4.6±2.0** | **8.3±3.0** | **13.6±4.0** | **9.9±6.0** |
| **Compressive modulus, kPa** | **212±44** | **125.0±39.0** | **112.0±16.0** | **94.0±19.0** | **113.0±22.0** | **139.0±27.0** |
