# Supplementary figures and images for: Chemical interactions in composites of gellan gum and bioactive glass: self-crosslinking and in vitro dissolution
Source: Front Chem. 2023 May 12;11:1133374. doi: 10.3389/fchem.2023.1133374 (PMC10213777; doi:10.3389/fchem.2023.1133374)

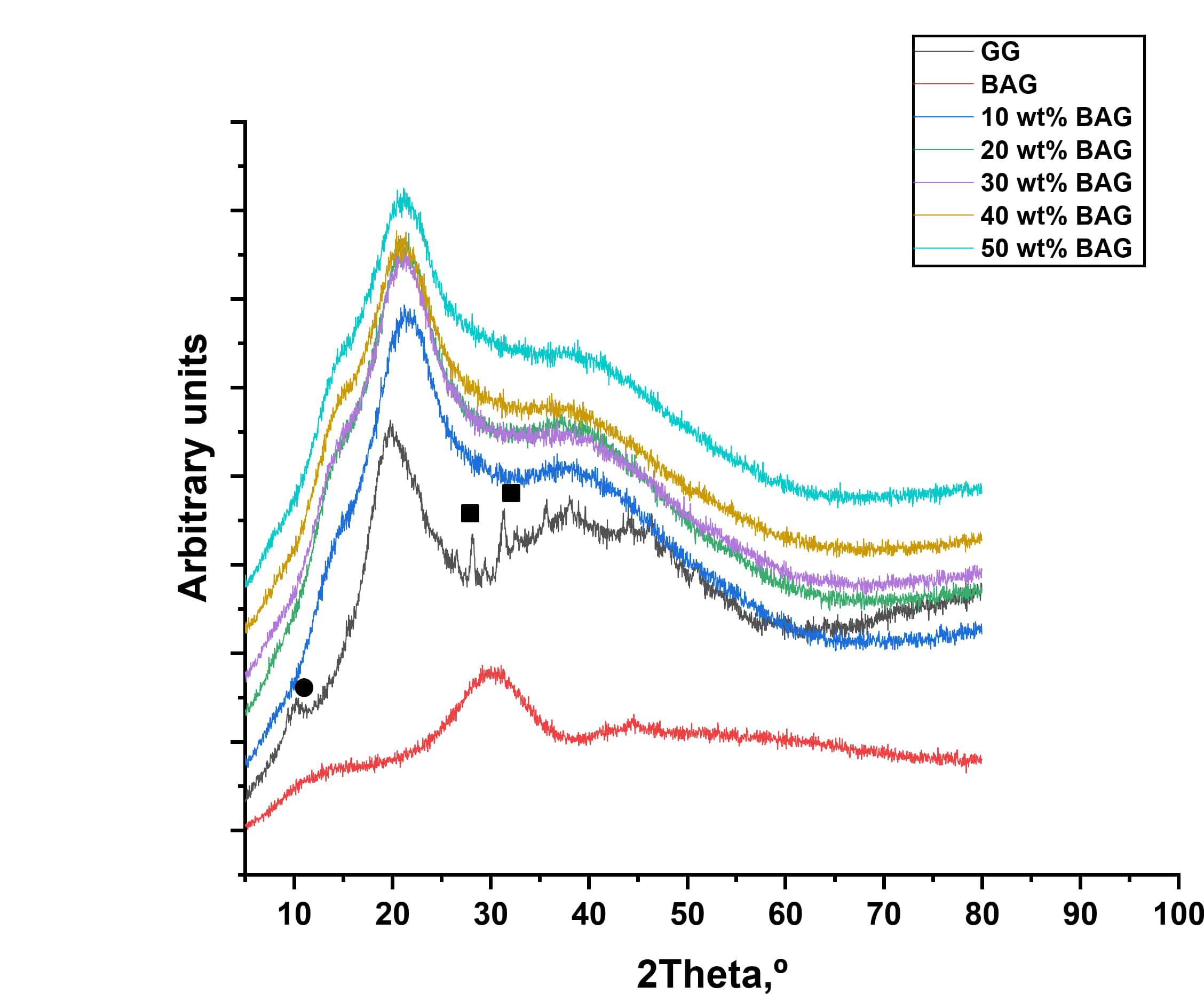

Supplement: Supplementary file 2 [file Image3.jpg]

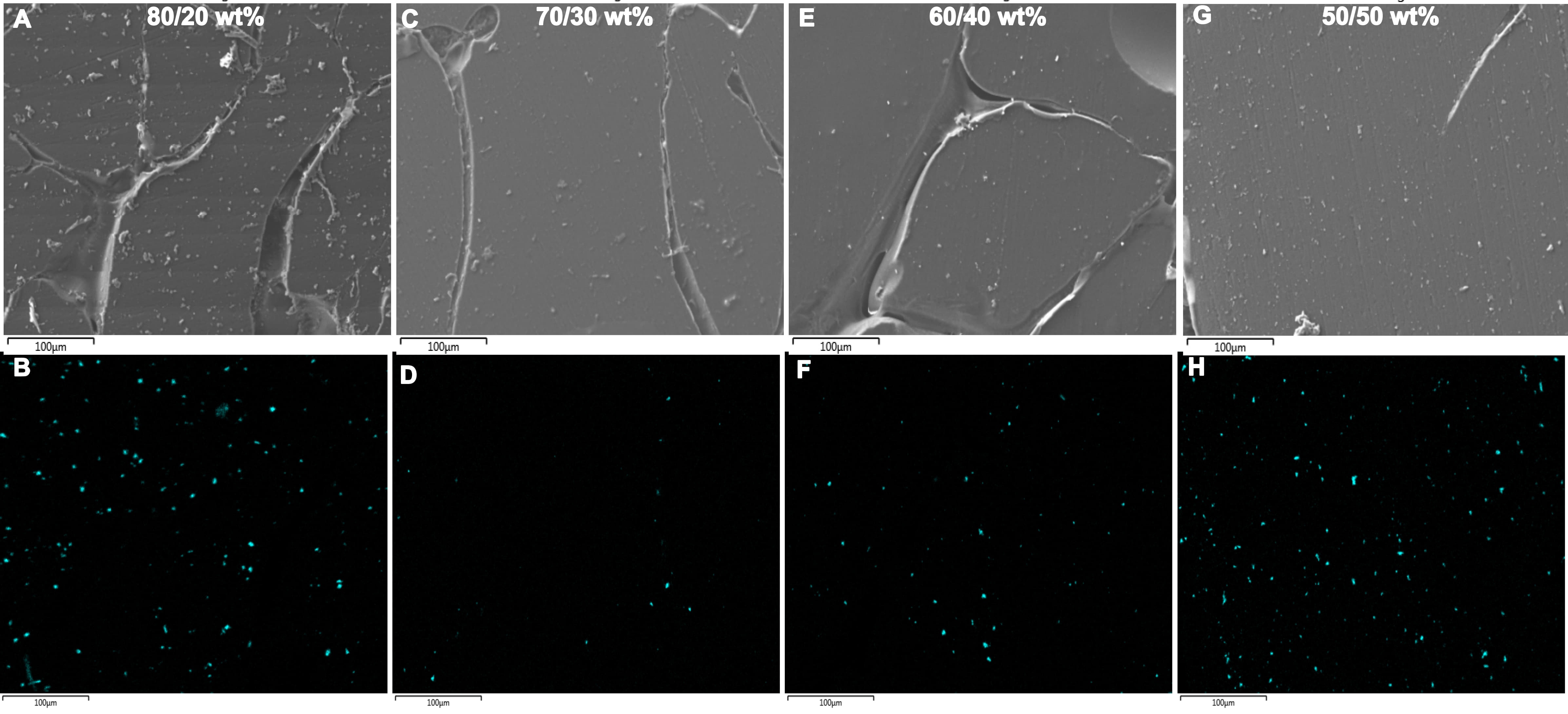

Supplement: Supplementary file 4 [file Image1.JPEG]

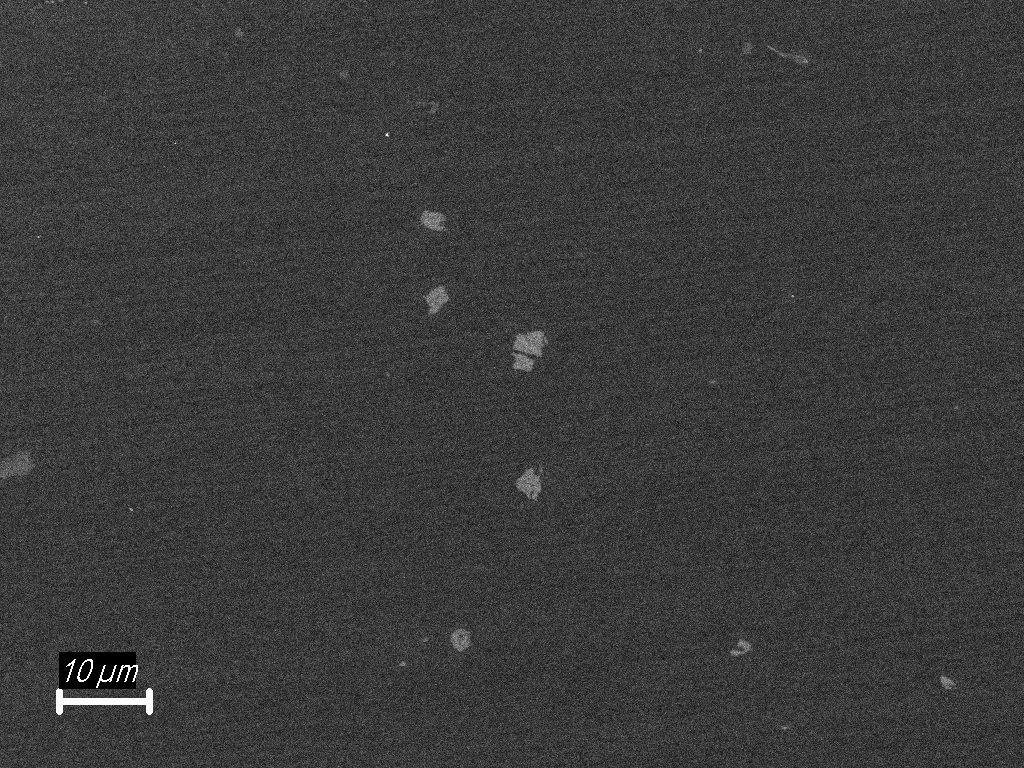

Supplement: Supplementary file 5 [file Image2.JPEG]
