# Supplementary material for: Chemical interactions in composites of gellan gum and bioactive glass: self-crosslinking and in vitro dissolution
Source: Front Chem. 2023 May 12;11:1133374. doi: 10.3389/fchem.2023.1133374 (PMC10213777; doi:10.3389/fchem.2023.1133374)
Supplement: Supplementary file 3 [file DataSheet3.docx]

| **GG/BAG, wt %** | **The remaining mass of the sample, %**  ***In vitro* dissolution in PBS, 1-14 days** | | | | | |
| --- | --- | --- | --- | --- | --- | --- |
|  | **Measured Day 0** | **Measured Day 1** | **Measured Day 2** | **Measured Day 3** | **Measured Day 7** | **Measured Day 14** |
| **90/10** | 100% | 87%±1 | 90%±1 | 107%±4 | 98%±5 | 96%±5 |
| **80/20** | 100% | 99%±1 | 98%±1 | 106%±2 | 105%±2 | 106%±5 |
| **70/30** | 100% | 97%±1 | 100%±1 | 106%±3 | 108%±2 | 110%±6 |
| **60/40** | 100% | 102%±1 | 99%±1 | 112%±6 | 105%±3 | 105%±5 |
| **50/50** | 100% | 99%±1 | 95%±2 | 108%±3 | 114%±4 | 109%±6 |

| **GG/BAG, wt %** |  | **The remaining mass of the sample, %**  ***In vitro* dissolution in Lysozyme, 1-48 hours** | | | | | | |
| --- | --- | --- | --- | --- | --- | --- | --- | --- |
|  | **Measured**  **0 hours** | **Measured**  **1 hour** | **Measured 2 hours** | **Measured**  **4 hours** | **Measured**  **6 hours** | **Measured 8 hours** | **Measured**  **24 hours** | **Measured**  **48 hours** |
| **90/10** | 100% | 91%±1 | 82%±3 | 79%±1 | 78%±1 | 84%±1 | 78%±1 | 78%±1 |
| **80/20** | 100% | 96%±1 | 94%±1 | 94%±1 | 88%±1 | 89%±1 | 91%±1 | 90%±1 |
| **70/30** | 100% | 98%±1 | 97%±2 | 94%±2 | 92%±2 | 89%±1 | 91%±1 | 93%±1 |
| **60/40** | 100% | 97%±1 | 94%±4 | 93%±1 | 92%±1 | 87%±4 | 94%±1 | 95%±1 |
| **50/50** | 100% | 97%±1 | 96%±4 | 95%±1 | 91%±1 | 93%±1 | 93%±1 | 95%±1 |

| **GG/BAG, wt %** |  | **The remaining mass of the sample, %**  ***In vitro* dissolution in PBS (as a control for Lysozyme), 1-48 hours** | | | | | | |
| --- | --- | --- | --- | --- | --- | --- | --- | --- |
|  | **Measured**  **0 hours** | **Measured**  **1 hour** | **Measured 2 hours** | **Measured**  **4 hours** | **Measured**  **6 hours** | **Measured 8 hours** | **Measured**  **24 hours** | **Measured**  **48 hours** |
| **90/10** | 100% | 95%±1 | 89%±1 | 84%±1 | 87%±1 | 83%±1 | 84%±1 | 85%±1 |
| **80/20** | 100% | 99%±1 | 97%±2 | 90%±1 | 92%±1 | 92%±1 | 94%±2 | 93%±2 |
| **70/30** | 100% | 95%±2 | 98%±1 | 83%±2 | 91%±2 | 88%±2 | 96%±1 | 93%±1 |
| **60/40** | 100% | 95%±1 | 98%±2 | 93%±1 | 91%±1 | 86%±1 | 96%±1 | 95%±1 |
| **50/50** | 100% | 95%±2 | 97%±1 | 87%±1 | 87%±2 | 85%±1 | 95%±2 | 95%±1 |

| **GG/BAG, wt %** | **pH of the supernatant**  ***In vitro* dissolution in PBS, 1-14 days** | | | | | |
| --- | --- | --- | --- | --- | --- | --- |
|  | **Measured Day 0** | **Measured Day 1** | **Measured Day 2** | **Measured Day 3** | **Measured Day 7** | **Measured Day 14** |
| **90/10** | 7.34 | 7.20±0.01 | 7.22±0.03 | 7.25±0.02 | 6.64±0.01 | 6.05±0.41 |
| **80/20** | 7.34 | 7.38±0.05 | 7.38±0.05 | 7.29±0.03 | 6.89±0.04 | 5.78±0.18 |
| **70/30** | 7.34 | 7.41±0.02 | 7.32±0.03 | 7.36±0.01 | 7.33±0.08 | 5.97±0.31 |
| **60/40** | 7.34 | 7.71±0.03 | 7.66±0.06 | 7.58±0.09 | 7.48±0.02 | 6.08±0.27 |
| **50/50** | 7.34 | 8.08±0.05 | 8.01±0.03 | 7.90±0.11 | 7.40±0.05 | 6.39±0.48 |

| **GG/BAG, wt %** | **pH of the supernatant**  ***In vitro* dissolution in Lysozyme, 1-48 hours** | | | | | | | |
| --- | --- | --- | --- | --- | --- | --- | --- | --- |
|  | **Measured**  **0 hours** | **Measured**  **1 hour** | **Measured 2 hours** | **Measured**  **4 hours** | **Measured**  **6 hours** | **Measured 8 hours** | **Measured**  **24 hours** | **Measured**  **48 hours** |
| **90/10** | 7.32 | 7.26±0.02 | 7.28±0.02 | 7.26±0.01 | 7.31±0.03 | 7.21±0.03 | 7.22±0.03 | 7.22±0.02 |
| **80/20** | 7.32 | 7.35±0.01 | 7.31±0.01 | 7.37±0.02 | 7.33±0.01 | 7.30±0.02 | 7.30±0.04 | 7.23±0.04 |
| **70/30** | 7.32 | 7.45±0.03 | 7.36±0.01 | 7.44±0.01 | 7.35±0.02 | 7.42±0.01 | 7.37±0.01 | 7.24±0.01 |
| **60/40** | 7.32 | 7.60±0.01 | 7.42±0.03 | 7.50±0.01 | 7.39±0.01 | 7.54±0.01 | 7.63±0.02 | 7.28±0.03 |
| **50/50** | 7.32 | 7.59±0.04 | 7.44±0.01 | 7.80±0.04 | 7.52±0.05 | 7.68±0.01 | 7.87±0.01 | 7.42±0.01 |

| **GG/BAG, wt %** | **pH of the supernatant**  ***In vitro* dissolution in PBS (as control for Lysozyme), 1-48 hours** | | | | | | | |
| --- | --- | --- | --- | --- | --- | --- | --- | --- |
|  | **Measured**  **0 hours** | **Measured**  **1 hour** | **Measured 2 hours** | **Measured**  **4 hours** | **Measured**  **6 hours** | **Measured 8 hours** | **Measured**  **24 hours** | **Measured**  **48 hours** |
| **90/10** | 7.34 | 7.33±0.01 | 7.27±0.01 | 7.24±0.01 | 7.23±0.01 | 7.26±0.01 | 7.20±0.01 | 7.22±0.02 |
| **80/20** | 7.34 | 7.40±0.02 | 7.38±0.01 | 7.33±0.03 | 7.39±0.03 | 7.33±0.04 | 7.38±0.03 | 7.38±0.01 |
| **70/30** | 7.34 | 7.45±0.01 | 7.51±0.01 | 7.44±0.01 | 7.45±0.01 | 7.44±0.01 | 7.41±0.01 | 7.32±0.01 |
| **60/40** | 7.34 | 7.59±0.01 | 7.65±0.04 | 7.61±0.02 | 7.63±0.02 | 7.57±0.02 | 7.71±0.02 | 7.66±0.01 |
| **50/50** | 7.34 | 7.67±0.01 | 7.70±0.01 | 7.75±0.01 | 7.74±0.01 | 7.73±0.01 | 8.08±0.01 | 8.01±0.02 |

| **GG/BAG, wt %** | **pH of the supernatant**  ***In vitro* dissolution in SBF, 1-7 days** | | | | |
| --- | --- | --- | --- | --- | --- |
|  | **Measured Day 0** | **Measured Day 1** | **Measured Day 2** | **Measured Day 3** | **Measured Day 7** |
| **90/10** | 7.40 | 7.39±0.01 | 7.37±0.01 | 7.31±0.03 | 7.11±0.01 |
| **80/20** | 7.40 | 7.39±0.02 | 7.37±0.04 | 7.38±0.01 | 7.25±0.04 |
| **70/30** | 7.40 | 7.39±0.01 | 7.38±0.03 | 7.38±0.02 | 7.37±0.01 |
| **60/40** | 7.40 | 7.42±0.03 | 7.38±0.01 | 7.41±0.01 | 7.36±0.01 |
| **50/50** | 7.40 | 7.44±0.01 | 7.39±0.02 | 7.45±0.01 | 7.40±0.03 |
