# Supplementary material for: Chemical interactions in composites of gellan gum and bioactive glass: self-crosslinking and in vitro dissolution
Source: Front Chem. 2023 May 12;11:1133374. doi: 10.3389/fchem.2023.1133374 (PMC10213777; doi:10.3389/fchem.2023.1133374)
Supplement: Supplementary file 6 [file DataSheet2.docx]

**Supplementary material S2**

**Calculations on:**

1. **Mass of BAG per sample (m_BAG_/sample)**
2. **Volume the BAG occupies per sample (V_BAG_/sample)**
3. **Amount of particles per each condition (GG/BAG wt%)**

**For instance :**

**V_BAG_ V_sample_ , % calculated (mm^3^**/ **mm^3^) for 50/50wt% GG/BAG:**

**Input values:**

for m_BAG_ in sample

V_sample_ = 0.65 ml

V_batch_ = 4.5 ml

C_(Gellan Gum)_ = 5mg/ml

for V_BAG_/V_sample_

diameter of sample **(d_sample_) = 6.65 mm**

height of sample **(h_sample_)** **= 11.49 mm**

density of bioactive glass* **(Þ_BAG_) = 2.6 g/sm^3^**

mass of the glass in sample **(m_BAG_50/50) = 2.95 mg**

diameter of one bioactive glass particle **(d_1BAG_*) = 12 μm**

**BAG – bioactive glass*

**1BAG – one bioactive glass particle*

1. Calculations (mass of BAG per sample), mg

Once we know that concentration of GG is 5 mg/ml, then we can calculate amount of GG per 0.65 ml of sample or per one sample:

m (GG per sample) = V_sample_ * C_GG_ = 0.65*5 = 3.25 mg

Once we know the weight ratio of gellan gum and bioactive glass, we can estimate amount of glass per sample from the proportion:

$$m_{\frac{BAG}{sample}}= \frac{Weight ratio \frac{BAG}{sample} *m \frac{GG}{sample}}{weight ratio \frac{GG}{sample}}$$

**GG/BAG 80/20 wt%:** BAG per sample = 20*3.25/80 = 0.8125 **≈ 0.81**

| **GG/BAG, wt%** | **90/10** | **80/20** | **70/30** | **60/40** | **50/50** |
| --- | --- | --- | --- | --- | --- |
| **BAG per sample, mg** | **0.36** | **0.81** | **1.39** | **2.17** | **3.25** |

2. Calculations (V_BAG_ /V_sample_, %) and calculated changed of particles (N):

1. **V_sample_**

Volume of sample is calculated as volume of cylinder:

**V_sample_ = πr^2^h = 3.14*(6.5/2)^2^*11.5 = 398.87 mm^3^**

r = d/2

1. **V one BAG particle (V_1BAG_)**

Volume of one BAG particle is calculated as the volume of sphere: **V_1BAG_ = 4/3 πr³ = 9.04*10^-7^mm^3^**

diameter of one BAG particle (d_1BAG_*) = 12 μm = 12*10^-3^mm

r = d/2

1. **m one BAG particle (m_1BAG_)**

**m_1BAG_ = V_1BAG_* Þ_BAG_ = 9.04*10^-7^ * 2.6 = 2.35*10^-6^ mg**

Þ_BAG_ = 2.6 g/sm^3^ = 2.6*10^3^/10^3^ = 2.6 mg/mm^3^

1. **N (calculated amount of particles)**

Theoretical amount of particles are calculated as mass of glass into sample divided into mass of one bioactive glass particle.

For example: **GG/BAG 50/50wt%: N = 3.25/2.35*10^-6^ = 1 382 978**

| **GG/BAG, wt%** | **90/10** | **80/20** | **70/30** | **60/40** | **50/50** |
| --- | --- | --- | --- | --- | --- |
| **Number of calculated particles (N)** | **153 111** | **344 500** | **591 179** | **922 920** | **1 382 254** |

1. **V_BAG_**

Volume of bioactive glass is number of particles multiplied by the volume of one particle.

**GG/BAG 50/50 wt%: V _BAG_ = N*V_1BAG_ = 1 254 661*9.04*10^-7^ = 1.13 mm^3^**

1. **V_BAG_ /V_sample_**

From sections 1. and 5. we know the volume of BAG and volume of sample, thus:

**GG/BAG 50/50wt%: V_BAG_/V_sample_ = (1.13/398.87)*100% = 0.28%**

| **GG/BAG, wt%** | **90/10** | **80/20** | **70/30** | **60/40** | **50/50** |
| --- | --- | --- | --- | --- | --- |
| **V _BAG_/V _sample_ (calculated)** | **0.04** | **0.08** | **0.14** | **0.21** | **31** |

Calculated data :

| GG/BAG, wt% | V_sample_ | V_1BAG_ | m_1BAG_ | N | V _BAG_ | V_sample_ | V _BAG_/V_sample_*100% |
| --- | --- | --- | --- | --- | --- | --- | --- |
| 90/10 | 398.8714271 mm^3^ | 9.04x10^-7^ | 2.35x10^-6^ | 153111 | 0.14mm^3^ | 398.87mm^3^ | 0.04% |
| 80/20 |  |  |  | 344500 | 0.31mm^3^ |  | 0.08% |
| 70/30 |  |  |  | 591179 | 0.54mm^3^ |  | 0.14% |
| 60/40 |  |  |  | 922920 | 0.84mm^3^ |  | 0.21% |
| 50/50 |  |  |  | 1382254 | 1.25mm^3^ |  | 0.31% |

Measured data (µCT, 5.64pix, Figure 1):

Day 0

| GG/BAG, wt% | V_sample_ | V_BAG_ | V _BAG_/V_sample_*100% |
| --- | --- | --- | --- |
| 90/10 | 8.63x10^1^mm^3^ | 1.09x10^-4^mm^3^ | 0.00 |
| 80/20 | 1.34 x10^2^mm^3^ | 2.59 x10^-3^mm^3^ | 0.00 |
| 70/30 | 1.34 x10^2^mm^3^ | 9.25 x10^-2^mm^3^ | 0.07 |
| 60/40 | 1.34x10^2^mm^3^ | 2.52 x10^-1^mm^3^ | 0.19 |
| 50/50 | 1.28x10^2^mm^3^ | 4.04x10^-11^mm^3^ | 0.32 |

Day 7(*in vitro*, SBF)

| GG/BAG, wt% | V_sample_ | V_BAG_ | V _BAG_/V_sample_*100% |
| --- | --- | --- | --- |
| 90/10 | 1.10x10^2^mm^3^ | 3.01 x10^-4^mm^3^ | 0.00 |
| 80/20 | 1.06 x10^2^mm^3^ | 3.30 x10^-4^mm^3^ | 0.00 |
| 70/30 | 1.34 x10^2^mm^3^ | 3.49 x10^-4^mm^3^ | 0.00 |
| 60/40 | 1.34 x10^2^mm^3^ | 3.18 x10^-2^mm^3^ | 0.02 |
| 50/50 | 1.34 x10^2^mm^3^ | 5.51 x10^-3^mm^3^ | 0.00 |
