# Supplementary material for: Chemical interactions in composites of gellan gum and bioactive glass: self-crosslinking and in vitro dissolution
Source: Front Chem. 2023 May 12;11:1133374. doi: 10.3389/fchem.2023.1133374 (PMC10213777; doi:10.3389/fchem.2023.1133374)
Supplement: Supplementary file 7 [file DataSheet1.docx]

**Supplementary material S1**


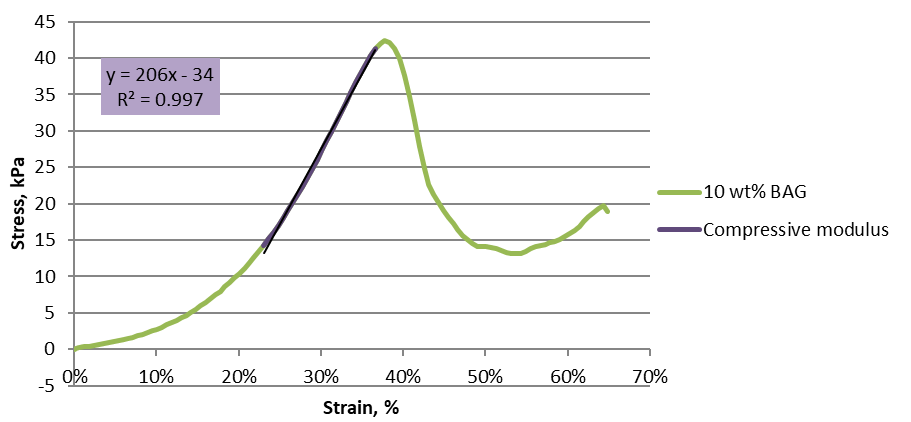


**A.** Representative stress-strain curve of 90/10 wt% GG/BAG samples with a highlighted elastic region from which compressive modulus 205 kPa was calculated. Fracture strength 42 kPa at 38 % strain.
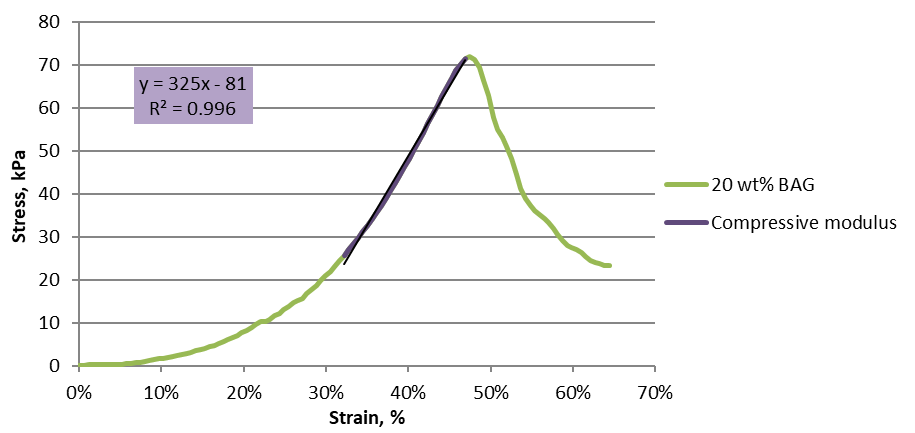


**B.** Representative stress-strain curve of 80/20 wt% GG/BAG samples with highlighted elastic region from which compressive modulus 325 kPa was calculated. Fracture strength 71 kPa at 48 % strain.


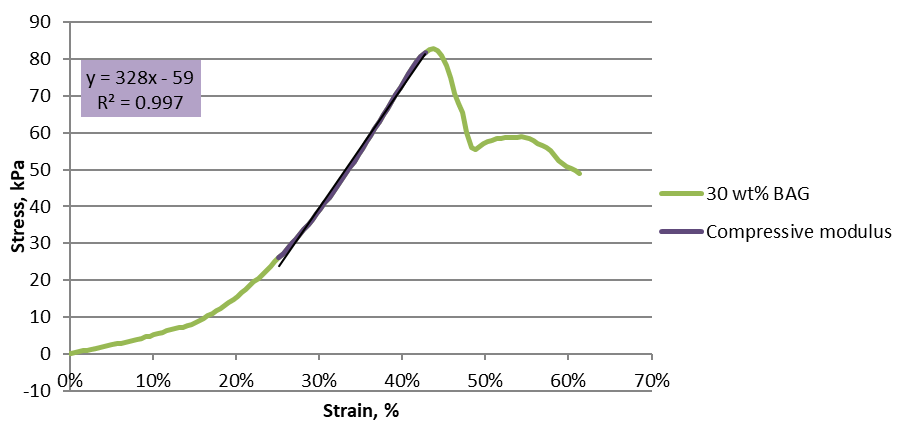


**C.** Representative stress-strain curve of 70/30 wt% GG/BAG samples with highlighted elastic region from which compressive modulus 328 kPa was calculated. Fracture strength 82 kPa at 44 % strain.


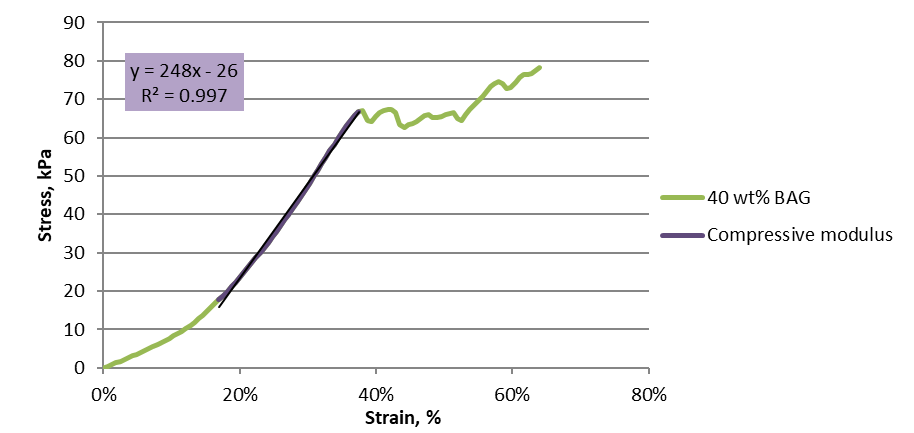


**D.** Representative stress-strain curve of 60/40 wt% GG/BAG samples with highlighted elastic region from which compressive modulus 248 kPa was calculated. Fracture strength 64 kPa at 39 % strain


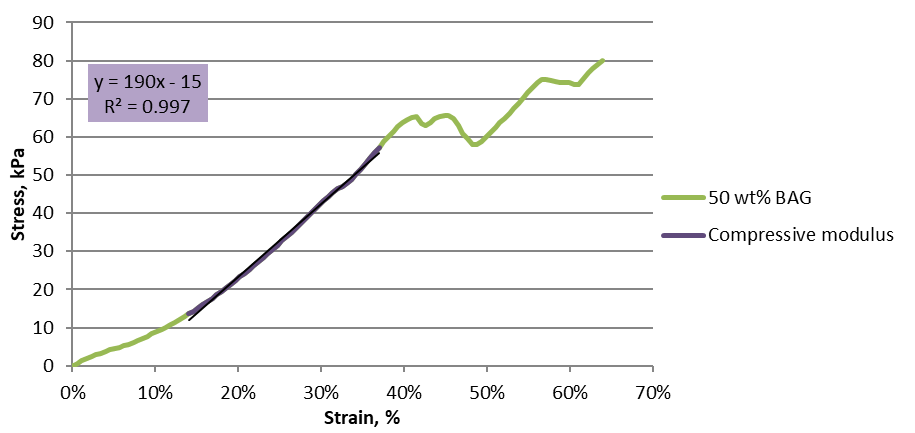


**E.** Representative stress-strain curve of 50/50 wt% GG/BAG samples with highlighted elastic region from which compressive modulus 190 kPa was calculated. Fracture strength 65 kPa at 45 % strain.
